# A short peptide derived from late embryogenesis abundant proteins enhances acid tolerance in *Escherichia coli* via modulation of two‐component regulatory systems

**DOI:** 10.1111/febs.70268

**Published:** 2025-09-27

**Authors:** Khaled Metwally, Shinya Ikeno

**Affiliations:** ^1^ Department of Biological Functions Engineering, Graduate School of Life Science and Systems Engineering Kyushu Institute of Technology Kitakyushu Japan; ^2^ Department of Genetics, Faculty of Agriculture Ain Shams University Cairo Egypt

**Keywords:** acid stress tolerance, differentially expressed genes (DEGs), *Escherichia coli*, LEA peptides, molecular docking simulation, RNA sequencing, two‐component regulatory systems

## Abstract

Late embryogenesis abundant (LEA) proteins are responsible for facilitating tolerance to various environmental stresses across diverse organisms. Group 3 LEA proteins are characterised by the presence of 11‐mer amino acid motifs, which inspired the design of short peptides with similar protective functions. Here, we designed a LEA peptide variant (LEA‐K) and evaluated its acid tolerance capacity in *Escherichia coli* BL21 (DE3) at pH4. Expression of LEA‐K peptide improved the bacterial viability under acidic stress, suggesting its protective functions. To explore the molecular mechanism of such tolerance, we combined the RNA‐sequencing (RNA‐Seq) technique and molecular docking simulations. Transcriptome analysis identified 283 differentially expressed genes (DEGs), and revealed metabolic reprogramming and activation of stress‐related pathways, including proton pumping, biofilm formation, and stress responsive systems. Functional enrichment analysis suggested a key role of two‐component regulatory systems (TCSs) such as reactive chlorine species (RCS), sensor histidine kinase BtsS/transcriptional regulatory protein BtsR, and DNA‐binding dual transcriptional regulator OmpR/sensor histidine kinase EnvZ. Protein–peptide docking simulations indicated potential interactions between LEA‐K and these TCSs, suggesting a mechanistic basis of the observed transcriptional modulation. These findings propose previously unknown functional roles for LEA peptides, not only acting as molecular shields but also as signal‐transducing modulators. This work expands our understanding of stress tolerance mechanisms and presents a new avenue for engineering stress‐resilient bacterial systems.

## Graphical abstract

Expression of the LEA‐K peptide in *E. coli* BL21 (DE3) improved bacterial viability under acidic stress (pH4), suggesting a protective function. RNA‐sequencing of LEA‐K‐expressing vs. non‐expressing cells revealed 283 differentially expressed genes (DEGs). In silico docking linked LEA‐K to two‐component systems (TCS) proteins (Rcs, BtsS/R, and EnvZ/OmpR) with favourable binding energetics, proposing a mechanistic basis for the transcriptomic changes and resilience to acidic stress.

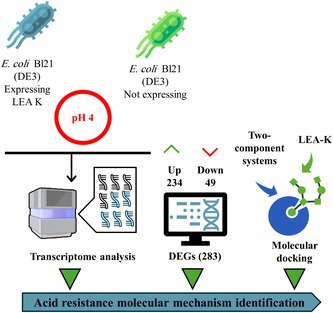

**Abbreviations**
ABC: ATP‐binding cassette
APC: amino acid‐polyamine‐organocation
AR: acid resistance
BP: biological process
CC: cellular component
CFU: colony‐forming units
CSP: cold shock protein
DEGs: differentially expressed genes
GABA: γ‐aminobutyric acid
GAD: glutamate decarboxylase
GO: gene ontology
GSA: glutamate‐γ‐semialdehyde
HSP: heat shock protein
IPTG: isopropyl‐β‐d‐thiogalactopyranoside
LEA: late embryogenesis abundant
MCode: molecular complex detection
MF: molecular function
NCS1: nucleobase:cation symporter −1
P5CDH: pyrroline‐5‐carboxylate dehydrogenase
PGA: poly‐β‐1,6‐*N*‐acetylglucosamine
PPI: protein–protein interaction
PRODH: proline dehydrogenase
PTS: phophoenol pyruvate:sugar phosphotransferase
Rcs: reactive chlorine species
RNA‐Seq: RNA‐sequencing
ROS: reactive oxygen species
RT‐qPCR: reverse transcription‐quantitative polymerase chain reaction
STRING: search tool for the retrieval of interacting genes/proteins
TCS: two‐component system
TF: transcription factor
TRAP: tripartite ATP‐independent periplasmic

## Introduction

Environmental stressors such as heat, cold, salinity, drought, ultraviolet radiation, and inappropriate pH levels constitute significant challenges to the survival and production of all living organisms [1]. This could result in declining global food production, which is a critical issue as the world's population continues to rise, driving the demand for sustainable and efficient food systems [2].

Among these, acidic pH represents a particularly severe environmental challenge that disrupts cellular homeostasis and impairs all biological systems [3]. This is evident not only in the agricultural ecosystems [3, 4], but also in industrial microbial fermentations, where acid stress can impair the growth and functionality of microorganisms essential for production, thereby reducing the efficiency of the valuable bio‐product yields [5, 6].

Gram‐negative bacteria such as pathogenic strains of *Escherichia coli* face harsh acidic environments, particularly in the gastrointestinal tract [7]. These deleterious conditions decline microbial viability by disrupting membrane integrity, impairing enzyme activity, and altering intracellular pH homeostasis [8]. Meanwhile, these pathogens could adapt by employing robust acid resistance mechanisms, including the two‐component regulatory systems (TCSs) and Acid Resistance (AR) pathways designated AR1 through AR6 which modulate gene expression, thereby mitigating the pH‐induced damage [9].

In contrast, *E. coli* BL21 (DE3), a commonly used strain in biotechnology, is susceptible to acidic stress due to the lack of native acid resistance mechanisms [10].

Late embryogenesis abundant proteins (LEA), firstly identified in plants [11, 12], have been shown to play pivotal roles in protecting cells against various environmental stressors such as desiccation [13, 14], high and low temperatures, UV, drought and salinity. Interestingly, LEA proteins have also been identified in bacteria, fungi and animals [15]. These proteins are intrinsically disordered and are believed to exhibit protective functions through mechanisms such as stabilising macromolecules, scavenging reactive oxygen species, and maintaining cellular homeostasis under stress conditions [16, 17].

Group 3 LEA proteins of the African sleeping chironomid *Polypedilum vanderplanki* have been associated with desiccation tolerance [14] and are characterised by repetitive 11‐mer amino acid motifs [18]. Prior research demonstrated that short synthetic peptides derived from these motifs strongly contributed in conferring stress resistance against heat, cold, salinity [19], UV [20] and acid stress [21] in *E. coli*. However, the detailed molecular mechanisms by which these LEA‐derived peptides confer acid tolerance remain unclear.

This study aims to: (a) evaluate the acid tolerance conferred by an expressed LEA‐K peptide, a LEA peptide variant designed from our prior work [21] in transformed *E. coli* BL21 (DE3) under pH4 conditions; (b) identify associated transcriptional changes using RNA‐Seq approach; and (c) explore potential interactions between LEA‐K and acid‐related two‐component systems using in silico protein–peptide docking. This research addresses a novel investigation for the mechanistic role of LEA peptides in bacterial acid stress tolerance and proposes their interaction with two‐component regulatory systems as a potential mode of action. Furthermore, the findings of this research have the potential to offer significant insights into the engineering of stress‐resistant microbial strains for industrial and environmental applications.

## Results and discussion

### Effect of LEA peptide expression on viability in low pH

**Fig. 1:**
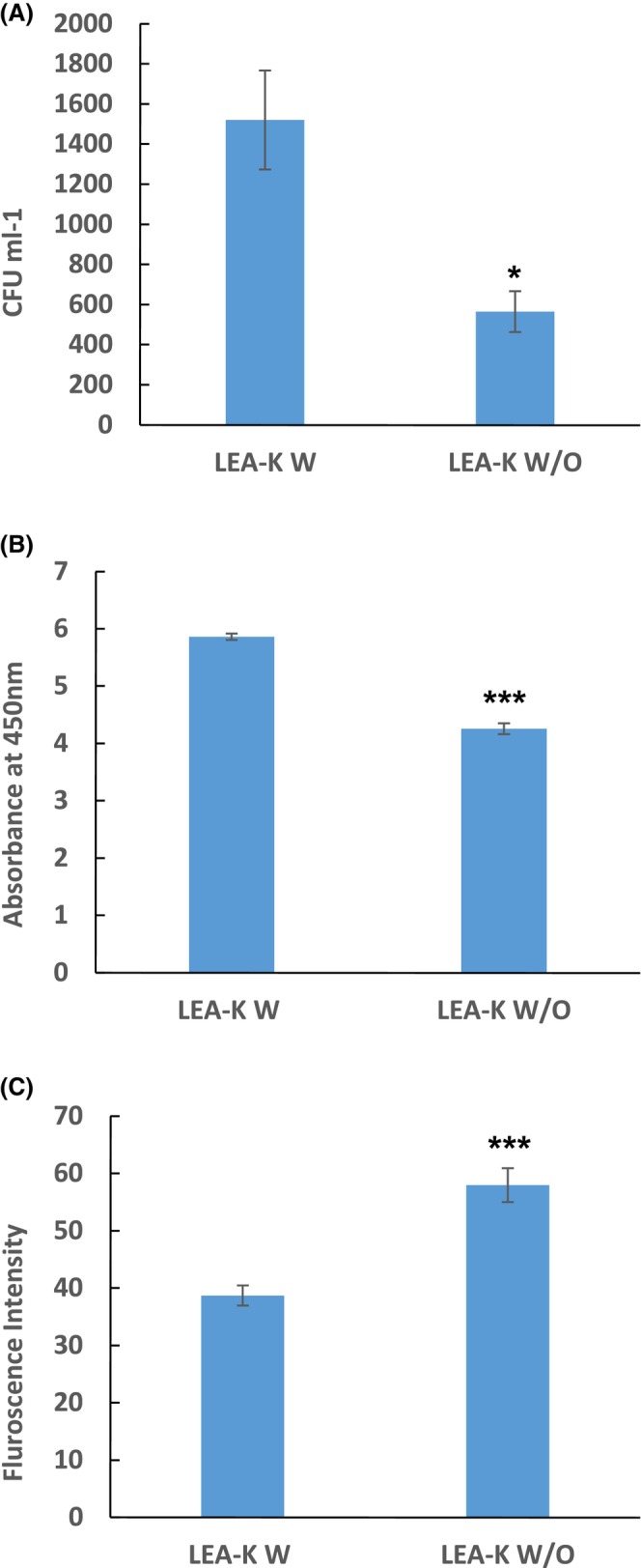
Growth capacity and reactive oxygen species (ROS) quantification of *E. coli* BL21‐ (DE3) expressing LEA‐K peptide in comparison to the control *E. coli* BL21 (DE3) not expressing LEA‐K. Both were grown in Luria‐Bertani (LB) medium at pH4, expression was induced with 0.1 mm isopropyl‐β‐d‐thiogalactopyranoside (IPTG) for the *E. coli‐expressing* LEA‐K (LEA‐K W), and 0 mm IPTG (no induction) for the control (LEA‐K W/O). (A) Colony‐forming unit (CFU)·mL^−1^ using the colony‐counting technique, (B) Growth capacity based on the WST formazan colorimetric change measured by microplate reader at 450 nm, (C) Total intracellular ROS measured by fluorescence intensity of DCFH‐DA using the fluorescence microplate reader. Data represent the mean (±SD) of five independent experiments (biological replicates), with three technical replicates each. Error bars indicate standard deviation. Statistical significance was determined by *t*‐test: *P < 0.05, ***P < 0.001.

Colony‐forming unit (CFU) data for cultures subjected to pH 4 acid stress are shown in Fig. 1A, illustrating distinct growth capacities between experimental groups. *E. coli* BL21‐ (DE3) expressing the LEA‐K peptide exhibited markedly improved growth, and recorded 1.52 × 10^7^ CFU·mL^−1^, compared to the control (0 mm Isopropyl‐β‐d‐thiogalactopyranoside (IPTG), non‐induced), whereas the control showed a 63% decrease in viability (5.65 × 10^6^ CFU·mL^−1^). These results are aligned with our previous findings [21], confirming that LEA‐K expression enhances the growth capacity under acidic stress. Our observations are also supported by prior studies [14, 15], demonstrating the essential roles of LEA proteins in conferring abiotic stress tolerance. Additionally, based on the reduction of WST to formazan which reflects the metabolic activity, LEA‐K‐expressing bacteria showed a higher absorption at 450 nm with 1.5 fold compared with the control (0 mm IPTG, non‐induced) as illustrated in Fig. 1B. These results highlight the potential utility of LEA peptide in developing acid resistant microbial strains through genomic integration strategies, particularly to mitigate the inhibitory effects of acidic byproducts such as acetate or lactate during industrial fermentation. This approach could prove especially valuable for organic acid (e.g. succinate) [22] and biofuels (e.g. ethanol and butanol) [23]. Furthermore, LEA peptides appear to improve recombinant protein expression under stress conditions. Studies demonstrate that co‐expressing LEA peptides with target proteins in *E. coli* can boost protein yields, offering a practical strategy for industrial‐scale enzyme and metabolite production [24].

### Total ROS quantification

The intracellular ROS accumulation strongly contributed to the osmotic stress and acidic stress [25]. Data illustrated in Fig. 1C showed that *E. coli* BL21 (DE3) not expressing LEA‐K exhibited 1.5 fold in the total quantified intracellular ROS compared with that expressing LEA‐K, which remains in the normal values recorded at pH7 (data not shown), reflecting a 33% reduction of total ROS in LEA‐K‐expressing bacteria. This result suggests that LEA‐K‐expressing bacteria has activated some mechanisms through which it could overcome the accumulation of ROS.

### Sequencing output and alignment

RNA samples from three biological replicates of pH4 stress subjected *E. coli* BL21 (DE3) treatment (LEA‐K‐expressing) and control (not expressing) groups were used for Illumina Genome Analyzer deep sequencing. Approximately 90 million and 82 million raw reads were generated for the LEA‐K‐expressing and control samples, respectively. After quality filtering, the clean read numbers were reduced to 89 million for the treatment group and 81 million for the control group. In total, 173 million raw reads and 170 million cleaned reads were obtained (Table 1). Over 96% of the clean reads had Phred quality scores above Q20. Obtained clean reads were mapped to the reference genome of *E. coli* BL21 (DE3) using bowtie2 software, generating a mapping efficiency of 86.66% and 87.67% for the LEA‐K‐expressing samples and controls, respectively, indicating the high score quality alignment required for downstream transcriptomic analysis.

**Table 1:** Summary of the sequencing results.

Treatment	Condition	Raw reads	Clean reads	Total mapped
LEA‐K 0.1 mm1	Treatment (expressing LEA‐K)	31 553 544	31 155 801	26 663 134 (85.58%)
LEA‐K 0.1 mm2	Treatment (expressing LEA‐K)	29 595 186	29 162 179	25 534 403 (87.56%)
LEA‐K 0.1 mm3	Treatment (expressing LEA‐K)	29 701 039	29 298 843	25 364 008 (86.57%)
	Subtotal	90 849 769	89 616 823	77 561 545 (86.54%)
LEA‐K 0 mm1	Control (not expressing LEA ‐K)	29 793 168	29 402 627	26 724 047 (90.89%)
LEA‐K 0 mm2	Control (not expressing LEA ‐K)	27 453 852	27 117 975	23 581 791 (86.96%)
LEA‐K 0 mm3	Control (not expressing LEA ‐K)	24 973 199	24 660 275	21 005 622 (85.18%)
	Subtotal	82 220 219	81 180 877	71 311 460 (87.84%)
	Total	173 069 988	170 797 700	

### Differential expression analysis

**Fig. 2:**
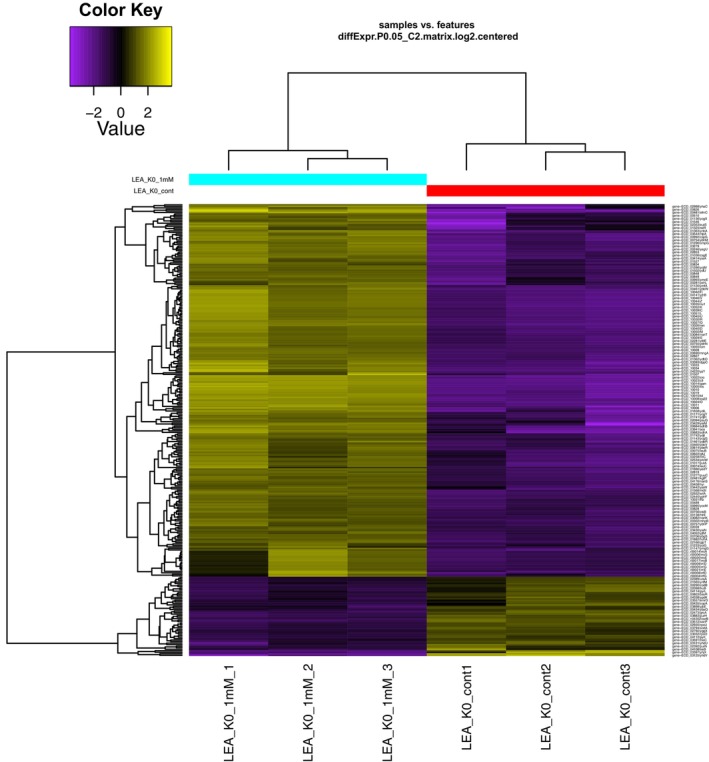
Hierarchical clustering analysis for the transcriptome of *E. coli* BL21 (DE3) control not expressing LEA‐K (LEA_K0_cont1, LEA_K0_cont2, LEA_K0_cont3) and *E. coli* BL21‐ (DE3) expressing LEA‐K (LEA_K0_1mm_1, LEA_K0_1mm_2, LEA_K0_1mm_3). All samples were grown in Luria‐Bertani (LB) medium at pH 4, *P*‐value < 0.005 and log2 (fold change) > 2.

Differentially expressed genes (DEGs) at the threshold of *P* < 0.005 and a log_2_ fold change > 2 were identified based on normalised FPKM values. Genes showing increased expression were considered upregulated while those showing decreased expression were classified as downregulated under acidic conditions (pH 4) in *E. coli* BL21‐ (DE3), expressing LEA‐K (0.1 mm IPTG) compared to the non‐expressing control (0 mm IPTG). A total of 283 DEGs were identified by both deseq2 and edger, comprising 234 upregulated and 49 downregulated genes (Table S1). Among these, six unigenes (2.1%) were exclusively expressed in LEA‐K‐expressing cells, whereas 97.9% of the unigenes were shared between both conditions (Table S2). Hierarchical clustering analysis based on log_10_‐transformed FPKM values from all six samples is presented in Fig. 2. In the heatmap, yellow bands indicate upregulated DEGs, while purple bands represent downregulated DEGs.

### Functional annotation of differentially expressed genes

Functional annotation was specifically carried out on the differentially expressed genes (DEGs) to elucidate the molecular mechanisms affected by LEA‐K expression under acidic stress. By narrowing the focus to these DEGs, this analysis highlights gene expression changes that contribute to stress adaptation and provides insight into the specific responses mediated by LEA‐K. Of the 283 DEGs, 52 (18.37%) were identified as hypothetical proteins, while approximately 81.63% were functionally annotated (Table S2A). As previously noted, six unigenes (2.1%) were exclusively expressed in LEA‐K‐expressing *E. coli*. Among these, three had unknown functions (Table S2). blast searches against the UniProt database [26] revealed that one of the uncharacterised genes encodes a transposase, while the other two remained unannotated. The remaining three LEA‐K‐specific unigenes were functionally annotated. One was *ybbV*, which encodes a transcription factor. The other two, *yjbL* and *yddK*, encode proteins with known or predicted regulatory roles. YjbL is considered a member of the response regulator family according to UniProt [26], while *yddK* encodes an outer membrane glycoprotein that may play a role in protein–protein interactions due to the presence of multiple leucine‐rich repeats (LRRs) in its structure [27]. A previous study reported the linkage between YddK expression and the pathogenicity of certain *E. coli* strains infecting humans [28]. Generally, the functional annotation of DEGs indicated that obtained DEGs were mostly involved in stress tolerance pathways, including DNA/RNA repair, chaperones, acid stress response, oxidoreductase activity, energy metabolism, and various transporter and antiporter systems.

### Genes involved in DNA/RNA repair and chaperones

Functional annotation of DEGs revealed 18 unigenes related to nucleic acid repair and chaperone activity. Among these, 16 genes were upregulated, while only two were downregulated (Table S1A). In *E. coli* BL21‐ (DE3) expressing LEA‐K, the *rec*T (also known as bet) gene, involved in DNA recombination‐based repair, was upregulated 3.78‐fold. During the acidic stress, this gene plays an important role in stress tolerance by facilitating DNA renaturation [10, 29]. Similarly, *yda*C (formerly *rcb*A), which contributes to chromosome integrity by reducing double‐strand break frequency, was upregulated 2.33‐fold [30]. The *ykf*J gene, encoding the RNA ligase RTCB, was upregulated 2.45‐fold. Under stress conditions, this enzyme catalyses RNA repair and tRNA splicing [31, 32]. Beyond nucleic acid repair, chaperone‐encoding genes were also upregulated. Notably, *hchA*, encoding the Hsp31 heat shock protein, exhibited a 2.1‐fold increase. Hsp31 is involved in the management of misfolded proteins under both high‐temperature and acidic stress conditions [33, 34, 35]. It also acts as a weak glyoxalase that detoxifies carbonyl compounds such as methylglyoxal accumulated under stress [36]. Additionally, the methionine sulfoxide reductase system (*msr*PQ) was activated, as shown by a 2.53‐fold upregulation of *yedY* (or msrPQ). This system repairs oxidised periplasmic proteins that accumulate under acid stress [37, 38]. Cold shock proteins *csp*G (also known as *yhi*D) and cspH were also upregulated 3.00‐ and 2.65‐fold, respectively. These proteins function as RNA chaperones that stabilise cellular RNA and support protein synthesis by preventing the formation of mRNA secondary structures under stress [39, 40].

The coordinated upregulation of these systems directly correlates with the observed acid tolerance phenotype in LEA‐K‐expressing bacteria. By safeguarding DNA from acid‐induced breaks (*rec*T, *yda*C), maintaining RNA functionality (*ykf*J, *csp*G/H), and preventing protein aggregation (*hch*A, *msr*PQ), LEA‐K enhances cellular viability under pH 4 stress, explaining its protective role at the molecular level.

### Genes involved in biofilm formation

Biofilms generate protective microenvironments that help bacteria to mitigate acidic stress by physically shielding cells, thus stabilising local pH gradients within the extracellular matrix [41]. In this study, we identified 18 unigenes associated with biofilm assembly and formation, of which 17 were upregulated and only one was downregulated (Table S1B). LEA‐K‐expressing *E. coli* BL21 (DE3) showed upregulation in genes *pgaA*, *pgaB*, *pgaC* and *pgaD* with 3, 2.74, 2.96 and 2.61‐fold change, respectively. These genes encode proteins involved in synthesis, processing and exportation of the poly‐β‐1,6‐*N*‐acetylglucosamine (PGA) required for biofilm formation [42, 43]. Their upregulation suggests a protective barrier against acid diffusion, directly contributing to the observed elevated viability at pH 4. Moreover, genes involved in the formation of proteinaceous micro‐compartments such as *eutS* showed also upregulation with a 2.30‐fold change in LEA‐K‐expressing bacteria. This gene encodes shell protein which is part of the ethanolamine‐degrading metabolosome structure [44]. Notably, *ymgA*, which encodes a connector protein within the RcsB–RcsC two‐component regulatory system, was upregulated by 2.34‐fold. This suggests that LEA‐K may activate the Rcs system, a known regulator of acid stress responses and biofilm formation [45, 46]. Curli fimbriae–associated genes (*csgE*, *sfmA*, *sfmC*, *sfmD*, *sfmH*) also showed substantial upregulation (2 to 4‐fold). These fimbriae are critical for adhesion and robust biofilm structure [41, 47]. Additional pilus‐related operons, including Yad (*yadK*, *yadM*) and Fim (*fimC*, *fimH*, *fimL*), were likewise elevated in LEA‐K‐expressing cells, supporting their role in surface attachment and survival in acidic environments [48, 49].

### Acid stress‐responsive genes

A total of nine unigenes associated with acid stress response were identified, of which six were upregulated and three were downregulated (Table S1C). In *E. coli‐expressing* LEA‐K, the *yagU* gene was upregulated 3‐fold. This gene encodes an inner membrane protein known to be induced by acid exposure [46]. Previous studies proposed its critical role under extreme acid conditions (pH 2), as they reported not only the significant increase in gene expression as the pH decreases, but also that mutant strains lacking *yagU* exhibited reduced acid resistance. However, the precise mechanism of action remains unclear [50, 51]. In addition, the *rclC* gene, encoding a membrane protein that confers resistance to reactive chlorine species (RCS) such as hypochlorous acid (HOCl), was upregulated by 2.43‐fold. This gene has been implicated in protecting proteins and membranes during oxidative stress, and its deletion increases sensitivity to HOCl [52, 53, 54].

Notably, *yjiY* (also known as BtsT) showed 3.25‐fold upregulation. It encodes an inner membrane protein that functions as a pyruvate:H^+^ symporter, facilitating proton‐coupled pyruvate transport under nutrient‐limiting conditions [55]. Conversely, *yhjX*, encoding a putative transporter of the major facilitator superfamily, was downregulated by 4.28‐fold. Both *yjiY* and *yhjX* are regulated by the BtsSR two‐component system [55]. The expression pattern—upregulation of *yjiY* and downregulation of *yhjX*—is characteristic of the acid resistance system 6 (AR6), recently identified in pathogenic *E. coli* strains [55, 56]. This pattern reflects a finely tuned regulatory mechanism that limits cytoplasmic acidification and promotes proton flux balance. The physiological role of *yhjX* remains debated: although previously hypothesised to function as a pyruvate efflux pump, recent studies suggest it may instead regulate extracellular pyruvate levels indirectly [57, 58]. In support of AR6 activation, additional oxidative stress response genes were differentially expressed, such as *sodA*—encoding manganese superoxide dismutase—was downregulated by 2.41‐fold. This enzyme detoxifies superoxide radicals and reactive oxygen species (ROS), suggesting that LEA‐K expression might activate alternative antioxidant pathways [25]. Likewise, *yhcN*, a gene strongly correlated with cytoplasmic acidification, was downregulated by 2.75‐fold [46, 59]. This suggests that LEA‐K‐expressing *E. coli* experiences less cytoplasmic acid stress, possibly due to active proton management or intracellular buffering systems.

Collectively, the aforementioned transcriptomic shifts indicated a multifaceted acid‐stress adaptation strategy in LEA‐K expressing *E. coli* through membrane stabilisation mediated by *yag*U gene, proton‐pyruvate coupling mediated by both *yji*Y and *yhj*X to counteract acidification, and mitigation of secondary oxidative stress mediated by *rcl*C. This regulatory network aligns with the observed phenotypic resilience, as LEA‐K appears to enhance both proton management and cellular integrity, thereby minimising acid‐induced cytotoxicity.

### Metabolism

Our analysis revealed identification of 61 unigenes involved in metabolism, of which 45 were upregulated and 16 were downregulated (Table S1D). Overall, *E. coli*‐expressing LEA‐K showed a reprogrammed metabolic shift including the activation of a glyoxylate cycle indicated by the increased expression of the *aceA* (isocitrate lyase) and *aceK* (malate synthase) genes, with fold changes of 3.40 and 3.04, respectively. It is well known that the glyoxylate cycle plays a crucial role in *E. coli* during acid stress through promoting energy conservation, thereby reducing acidification via bypassing the CO_2_‐releasing steps in TCA cycle [60, 61, 62]. Additionally, the gene *hyi*, encoding hydroxypyruvate isomerase, which is a key enzyme in glyoxylate metabolism and carbon redirection during stress [63] was upregulated by 2‐fold.

Similarly, the biotin synthesis pathway was also enhanced, indicated by the upregulation of the genes *bioB*, *bioC*, *bioD* and *bioF*, with fold changes ranging from 2.27 to 2.71. Biotin (vitamin B7) is a key cofactor for carboxylation reactions involving acetyl‐CoA carboxylase and pyruvate carboxylase, both of which are critical in fatty acid synthesis and central metabolism [64]. Previous studies have reported that the cellular demand for biotin increases during acid stress to support the glyoxylate cycle (via acetyl‐CoA carboxylase) and the TCA cycle (via pyruvate carboxylase), thereby maintaining metabolic flux, energy production and cellular integrity [64, 65].

LEA‐K‐expressing *E. coli* also exhibited a shift toward more efficient acetate recycling into central metabolism, evidenced by the 2.87‐fold upregulation of the *acs* gene. This gene encodes an enzyme that converts acetate into acetyl‐CoA, a key metabolite involved in energy generation, biosynthesis and metabolic flexibility. This observation aligns with the upregulation of *aceA* and *aceK*, which direct acetyl‐CoA into the glyoxylate cycle to bypass the TCA cycle under acid stress conditions [66, 67]. Furthermore, genes involved in the sialic acid catabolism pathway—*yjhB* (*nanX*), *kpsT*, *yjhC*, *nanA*, *nanC*, *nanM*, *nanS* and *nanT*—were upregulated, with fold changes ranging from 2.10 to 3.19. This indicates enhanced capacity to utilise sialic acid as an alternative carbon source, generating intermediates such as pyruvate that feed into energy and biosynthetic pathways during acid stress [68]. This metabolic pathway is commonly used by pathogenic *E. coli* to survive under the extreme acidic conditions of the human gastrointestinal tract [69, 70]. The proline utilisation pathway, mediated by the *putA* gene, was also upregulated by 2.58‐fold. *putA* encodes a bifunctional enzyme with proline dehydrogenase (PRODH) and pyrroline‐5‐carboxylate dehydrogenase (P5CDH) activities [71]. In this pathway, *putA* plays a key role in maintaining intracellular pH homeostasis, as the P5CDH domain oxidises glutamate‐γ‐semialdehyde (GSA) to glutamate using NAD^+^ as a cofactor—a reaction that consumes intracellular protons [72]. The resulting glutamate also functions as an osmoprotectant, enhancing acid stress tolerance [73]. In addition, the upregulation of *sdhA* and *sdhB* (2.64‐ and 3.24‐fold, respectively) in LEA‑K‐expressing *E. coli* indicates a metabolic adjustment to promote energy production, pH regulation and redox homeostasis. These genes encode subunits of succinate dehydrogenase, a key enzyme complex in both the TCA cycle and the electron transport chain, linking carbon metabolism with respiration. Their increased expression under acidic conditions implies enhanced oxidative phosphorylation and proton consumption during respiration [46, 74].

Differential expression analysis also revealed upregulation of *lacA*, *lacY* and *lacZ*, with fold changes of 3.16, 3.46 and 3.79, respectively, reflecting enhanced lactose metabolism. *lacA* encodes thiogalactoside transacetylase, which may assist in detoxifying non‐metabolisable sugar analogues, thereby preventing the accumulation of toxic byproducts. The proton‐coupled symport activity of *lacY* may further contribute to intracellular pH modulation [75]. Putrescine metabolism was also activated in LEA‐K‐expressing *E. coli*, as evidenced by the upregulation of *puuB* and *puuC* (2.2‐ and 2.4‐fold, respectively). The conversion of putrescine to γ‐aminobutyric acid (GABA) via the γ‐glutamyl cycle is an effective strategy under acidic stress, resulting in the release of ammonia and neutralisation of intracellular protons [76, 77]. The *puuC* gene encodes gamma‐glutamyl‐gamma‐aminobutyraldehyde dehydrogenase, which not only facilitates glycine betaine biosynthesis but also plays a role in the reversible oxidation of betaine aldehyde. Moreover, *puuC* has been reported to participate in the ornithine degradation pathway, which is closely linked to the acid resistance system 5 (AR5)—an ornithine‐dependent acid‐tolerant system [78, 79]. This suggests that LEA‐K expression may also activate AR5 as part of its acid resistance strategy. Additionally, LEA‐K‐expressing *E. coli* activated a proton‐pumping mechanism via upregulation of the *ydjJ* gene by 2.5‐fold. The *ydjJ* encodes an enzyme that catalyses the NAD^+^‐dependent oxidation of l‐threonine to 2‐amino‐3‐ketobutyrate, a proton‐consuming reaction that supports pH homeostasis [80]. Interestingly, fructose metabolism genes (*fruA*, *fruB* and *fruK*) were downregulated. Recent studies have suggested that fructose metabolism may increase reactive oxygen species (ROS) production [81], and its catabolism can also elevate organic acid byproducts such as pyruvate, lactate and acetate under stress conditions [74, 82]. This downregulation may reflect an adaptive shift away from ROS‐ and acid‐generating pathways in LEA‐K‐expressing cells.

Together, these metabolic changes demonstrate how LEA‐K expression helps *E. coli* adapt to acidic stress. By favouring pathways that minimise acid production such as the glyoxylate cycle and acetate recycling, it utilises alternative carbon sources involving sialic acid, and consumes excess protons via proline and putrescine breakdown. These adaptations allow cells to maintain stable energy levels and intracellular pH, explaining the high tolerance capacity of LEA‐K‐expressing bacteria and the lower ROS observed.

### Transporters and porins

Functional annotation analysis revealed the identification of 47 DEGs, of which 40 were upregulated and seven were downregulated (Table S1E). Generally, *E. coli*‐expressing LEA‐K showed higher expression levels for genes encoding components of ATP Binding Cassette (ABC) transporters along with ATP‐independent transporters and other transportation systems. The genes *proV* and *proW* were notably upregulated, 3.0 and 2.5‐fold, respectively. They encode components of the ABC transporter complex facilitating the uptake of glycine betaine and proline betaine, both of which are known for their osmoprotective properties, suggesting their contribution to acid stress tolerance [78, 83]. Additionally, the genes *sphX* and *pstA* were upregulated by 3‐ and 2‐fold, respectively. Both encode proteins that are components of the PstSACB ABC transporter complex, which plays an important role in inorganic phosphate uptake [84]. Previous studies demonstrated not only the importance of the Pst system in tolerating acidic stress—especially in pathogenic *E. coli* during gastric challenge—but also that deletion of the pst genes resulted in a lower survival rate compared with the wild type at pH 3 [85, 86]. Phosphate is essential for the production of ATP, which in turn fuels up acid resistance mechanisms, including proton pumps and transporters that help regulate intracellular pH homeostasis [87].

In the context of phosphate transporters, the genes *ynbA* and *glpT* showed an upregulation by 3.0 and 2.57‐fold, respectively. The *ynbA* gene encodes an inner membrane protein predicted to be a diacylglycerol choline‐phosphotransferase, which is involved in phospholipid biosynthesis and it was suggested to play a crucial role in maintaining cellular integrity and homeostasis during environmental stress [88, 89]. Expression of *ynbA* is activated by BglJ–RcsB following acid stress [90]. In contrast, the *glpT* gene encodes a glycerol‐3‐phosphate : phosphate antiporter that is part of the phosphate antiporter system in *E. coli*, involved in the uptake of glycerol‐3‐phosphate in exchange for inorganic phosphate [91]. A recent investigation indicated that the increased expression of *glpT* is primarily linked to acid stress in *E. coli*, implying its involvement in membrane repair processes in response to acid‐induced injury [92]. Other ABC transporters involved in glutamate/aspartate uptake, such as *gltK* which encodes periplasmic binding protein capturing glutamate or aspartate, and *gltJ* which encodes a transmembrane permease, were upregulated, with fold changes of 2.63 and 2.17, respectively. They both facilitate transport across the membrane during acid stress. The glutamate decaboxylase acid resistance system (GAD) relies mainly on glutamate. Through this system, intracellular protons were consumed by converting glutamate to γ‐aminobutyric acid (GABA). Consequently, the GABA is exported from the cell via an antiporter, helping maintain cytoplasmic pH [76, 77, 92, 93]. Moreover, the genes *mglA* and *mglC* showed upregulation of 3.28 and 2.38‐folds, respectively. These genes are part of the methyl‐galactoside ABC transporter system, which includes MglA (ATP‐binding protein), MglB (periplasmic substrate‐binding protein) and MglC (membrane‐spanning transporter). *mglC* encodes the integral membrane protein responsible for galactoside transport. Previous studies showed that MglA plays an effective role in environmental stress tolerance, especially oxidative stress [94, 95], by influencing the expression of more than 100 genes and proteins in *Francisella tularensis*. Deletion of *mglA* results in significantly reduced bacterial growth under oxidative stress.

On the other hand, ATP‐independent transporters were also activated in *E. coli* BL21‐ (DE3) expressing LEA‐K. Among these, the *mngA* and *yhiD* genes were upregulated by 2.77‐ and 3.00‐fold, respectively. Both genes exhibit dual functions. The *mngA* gene is involved in the phosphoenolpyruvate : sugar phosphotransferase system (PTS) in *E. coli*, through mediating the uptake and phosphorylation of sugars, particularly mannose [96]. Furthermore, it emphasises the uptake of manganese ions, which are crucial for combating oxidative stress [97]. Likewise, *yhiD* plays a dual role, through participating in magnesium ion uptake [98], and is also implicated in an acid resistance system 3 (AR3), a glutamate‐independent system. This system is activated under acidic conditions and provides protection by reducing the effects of intracellular acidification [99].

Other ATP‐independent transporters, such as *ybbW*, were upregulated by 3.14‐fold. Formerly known as *allW*, this gene is associated with allantoin transport and is essential for nitrogen uptake under nutrient‐limiting or stress conditions [100]. *allW* is a member of the Nucleobase : Cation Symporter‐1 (NCS1) family which is part of the Amino Acid–Polyamine–Organocation (APC) superfamily. Members of this group are typically involved in redox processes, and oxidative stress responses and detoxification. Accordingly, the *ybbW* is suggested to support redox balance and protect cells from oxidative damage during acid stress [100]. Further supporting the enhancement of nitrogen uptake, several genes involved in dipeptide and oligo peptide transport in *E. coli*, including *dppB*, *dppC*, *dppD*, *oppB* and *oppC*, were upregulated with 2.2 to 3.3‐fold changes. Their upregulated expression indicated the organism's metabolic adaptability and capacity to synthesise stress‐mitigating compounds through peptide utilisation [101].

Similarly, LEA‐K‐expressing *E. coli* showed upregulated gene expression levels of genes encoding channel proteins and porins, of which eight porin‐related genes were identified. Among them, *ompG* and *ompL* showed 2.9 and 2.35‐fold changes, respectively. The *ompG* gene encodes a monomeric porin known for its role in nutrient uptake and pH regulation [102]. Under acidic conditions, *ompG* may regulate ion flux, particularly protons, to stabilise the periplasmic pH. Research indicates that *ompG* is induced under stress to optimise nutrient uptake while maintaining homeostasis [102, 103]. Furthermore, LEA‐K‐expressing *E. coli* showed enhanced activity of ATP‐independent periplasmic TRAP (Tripartite ATP‐independent Periplasmic) transport systems, as evidenced by the upregulation of *yiaO*, *yiaM* and *yiaN*, with fold changes of 3.26, 3.00 and 2.00, respectively. This system is involved in the uptake of 2,3‐diketo‐l‐gulonate, with the YiaO protein specifically binding to this compound and also interacting with ascorbate [104]. Previous studies reported that during environmental stress, ascorbate and its derivatives can serve as cellular energy sources [105].

Conversely, the LEA‐K‐expressing *E. coli* downregulated the expression of the genes *xanP* and *copA*, with fold changes of −2.95 and −2.20, respectively. *xanP* is involved in proton motive force–dependent, high‐affinity transport of xanthine, which may contribute to acid tolerance [106]. This downregulation may indicate a shift in the acid stress tolerance mechanism mediated by LEA‐K‐expressing *E. coli* [107]. Similarly, the *copA* gene encoding a P‐type ATPase involved in copper efflux was reduced. This reduction may be explained due to the decrease of solubility and availability of copper during acid stress, which in turn reduces the need for efflux activity, which could explain the downregulation of *copA* [108].

The broad upregulation of transporters and porins in LEA‐K‐expressing *E. coli* directly supports its acid‐tolerant phenotype by importing osmoprotectants mediated by *pro*V and *pro*W as well as phosphate via *pst*A and *glp*T to maintain membrane integrity and energy levels. Moreover, supplying substrates for proton‐consuming systems such as GAD via *gltK* and *glt*J, and optimising porin function (*omp*G) for pH homeostasis.

### Transcription factors prediction

The complete set of differentially expressed genes were analysed for their potential roles as transcription factors (TFs) using the tool deeptfactor [109]. Among the upregulated DEGs six candidates were predicted to function as transcription factors with confidence score ranging between 0.9159 to 0.9985 (Table S1F). In contrast, only two candidates from the downregulated list were predicted to function as TFs with confidence scores 0.7167 and 0.9995 (Table S1F). Out of the six predicted upregulated TFs, four were identified as phage related genes including phage activator protein cII, T7 RNA polymerase, antitermination protein Q, and ECOLX Protein ORFa in retron Ec67, recording a fold change of 3.72, 2.22, 3.06 and 3.34, respectively. Their expression is likely associated with the LEA‐K expression system, which is driven by the T7 promoter in the pRSF‐duet vector.

The remaining two upregulated TFs included EcpR with 3.06‐fold change and prediction score (0.9985) and ycaN with 2.25‐fold change with prediction score (0.9977). EcpR (MatA) is a LuxR‐type transcription factor which acts as a positive regulator at transcriptional and post‐transcriptional levels, particularly regulating the expression of *mat* operon in *E. coli* K‐12. It also interferes with bacterial motility and flagellar synthesis [110], while YcaN was predicted to be a LysR‐type transcription factor which is likely involved in regulation of threonine and formate utilisation [93, 111]. Among the downregulated TFs, YbaQ showed a 2.16‐fold decrease in expression (score 0.9995). It belongs to the Xre transcription factors family and has been predicted to regulate genes associated with metabolism [111]. Transcription of *ybaQ* is known to be affected by conditions such as biofilm formation, heat stress and a glucose‐lactose shift [93]. Similarly, YiaG, another predicted Xre transcription factors family, was downregulated by 2.17‐fold (score 0.7167). It is involved in regulating genes related to protein export, and its transcription is also influenced by environmental stress, stringent response, and nutrient shifts such as the glucose‐lactose transition [112].

Overall, the host‐encoded TF changes directly contribute to LEA‐K's acid tolerance phenotype through two key mechanisms: EcpR‐mediated motility suppression conserving energy for essential stress responses, while YcaN‐driven activation of threonine metabolism enhances proton consumption (as evidenced by *ydj*J upregulation). Furthermore, the downregulation of nutrient‐responsive TFs (YbaQ/YiaG) shifts metabolic priorities from flexible growth to dedicated stress adaptation, mirroring the metabolic rewiring observed in LEA‐K‐expressing cells. This coordinated TF regulation explains the improved acid survival by optimising energy allocation and proton management under stress conditions.

While the transcriptomic analysis provided a systems‐level view of gene expression changes associated with the expression of LEA‐K peptide, it could not delineate the regulatory mechanisms controlling these genes, leaving the question of how this peptide modulates their expression unresolved.

### Gene enrichment analysis

**Fig. 3:**
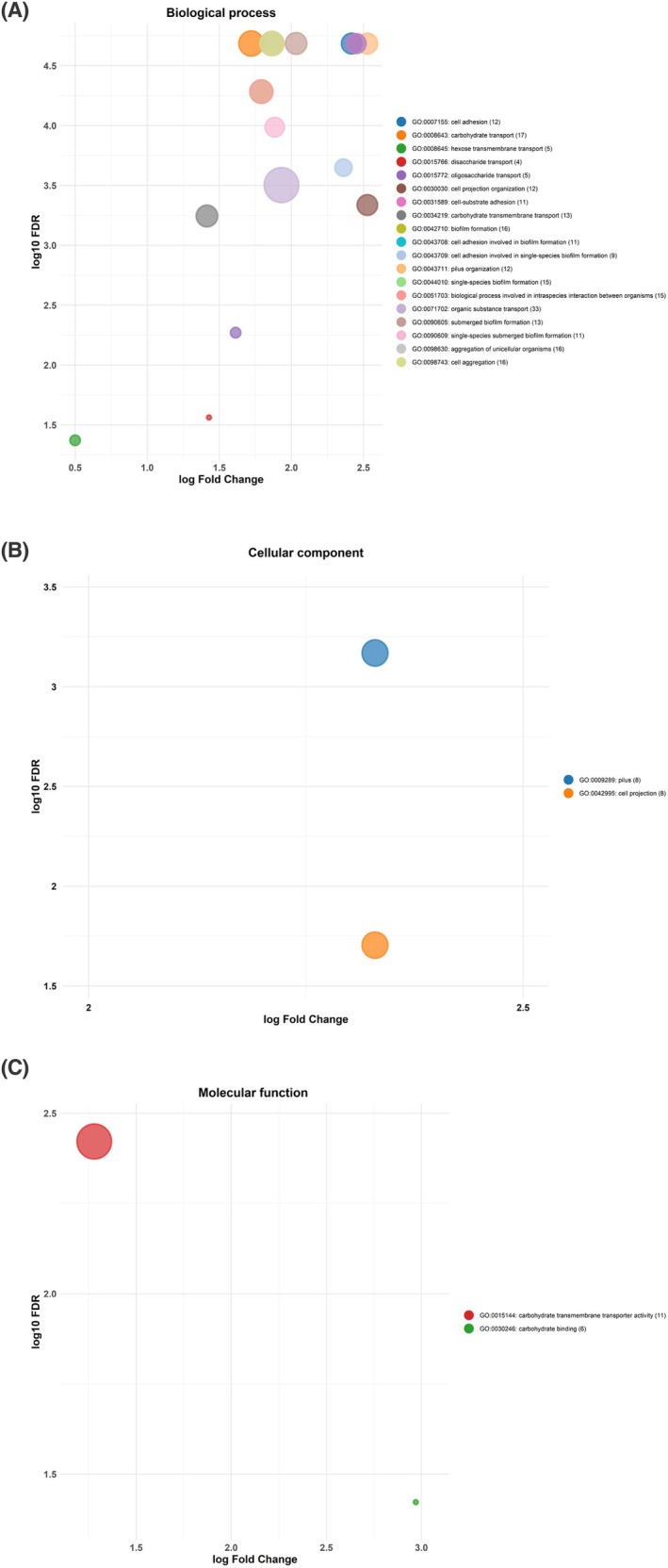
Bubble plot showing gene ontology (GO) classification of differentially expressed genes (DEGs), plotting expression changes (log2 fold change) against statistical significance (log10 false discovery rate [log10 FDR]). GO terms are grouped into three categories: (A) Biological process (BP), (B) Cellular component (CC), (C) Molecular function (MF). Bubble size corresponds to the number of genes (as indicated between brackets) per GO term, while colour distinguishes individual GO terms (see scale).

Clustering of the differentially expressed gene sets was performed based on GO terms into three main categories: biological process (BP), cellular component (CC), and molecular function (MF) as shown in Fig. 3 and Table S3A–C, respectively. Among these groups, the biological process category presents the most enriched proteins (246 in total). These proteins were involved in several functional clusters including submerged biofilm formation, pilus organisation, cell adhesion (particularly in relation to biofilm formation), and various active transport processes such as carbohydrate transport, organic substance transport, carbohydrate transmembrane transport, oligosaccharide transport, disaccharide transport, hexose transmembrane transport, and cell projection organisation. The molecular function category presents two clusters involving 17 enriched proteins, mainly related to carbohydrate transmembrane transporter activity and carbohydrate binding. Similarly, the cellular component category contained two main clusters—pilus and cell projection—comprising 16 enriched proteins.

### Protein network analysis

**Fig. 4:**
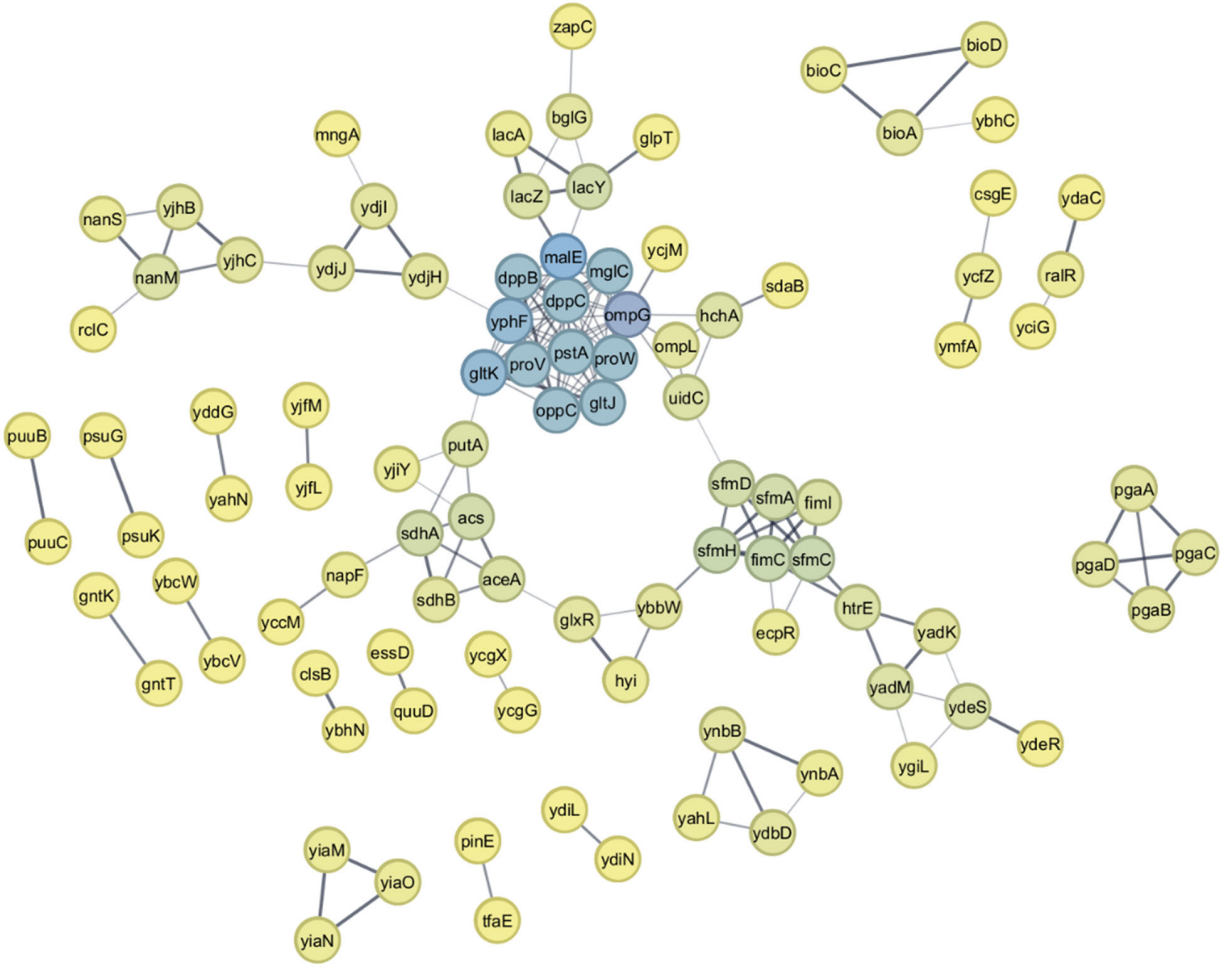
Protein–protein interaction network of upregulated genes under acidic pH (pH 4). The network contains 125 nodes (proteins) and 161 edges (interactions), with nine significant sub‐clusters identified using the MCODE algorithm (medium confidence; cutoff: 0.4). Node colors represent the degree of centrality (number of connections): blue indicates hub proteins with the highest connectivity (degree 11–15), green to yellow gradient shows moderately (degree 4–10) to weakly connected proteins (degree < 4), respectively.

The STRING (Search Tool for the Retrieval of Interacting Genes/Proteins) was employed to investigate the protein interaction networks underlying acid stress tolerance. STRING collects and integrates protein–protein interactions, including both physical interactions and functional associations [113]. The lists of upregulated and downregulated DEGs were uploaded to STRING for analysis. As illustrated in Fig. 4, the network analysis of upregulated genes revealed 125 nodes and 161 edges. The MCODE algorithm was applied to the resulting network and identified nine subclusters. Based on degree of centrality (i.e. connectivity), the central subcluster was visualised using blue nodes representing hub proteins with connectivity degrees ranging from 11 to 15 (Table S4A). This primary subcluster included the proteins OmpG, MalE, DppB, DppC, ProW, ProV, GltK, GltJ, PstA, YphF, MglC and OppC, all of which are transport‐related proteins. Their high connectivity compared to other subclusters, which ranged in colour from yellow to green, suggesting a central role in acid stress tolerance, as previously reported [78, 83, 101]. Another subcluster, characterised by degrees of centrality ranging from 4 to 5, comprised the proteins SfmA, SfmC, SfmD, SfmH, FimC, FimL and EcpR (MatA), which are believed to be associated with biofilm formation and fimbrial gene expression—processes that have been strongly linked to acid stress response [48, 49, 110].

**Fig. 5:**
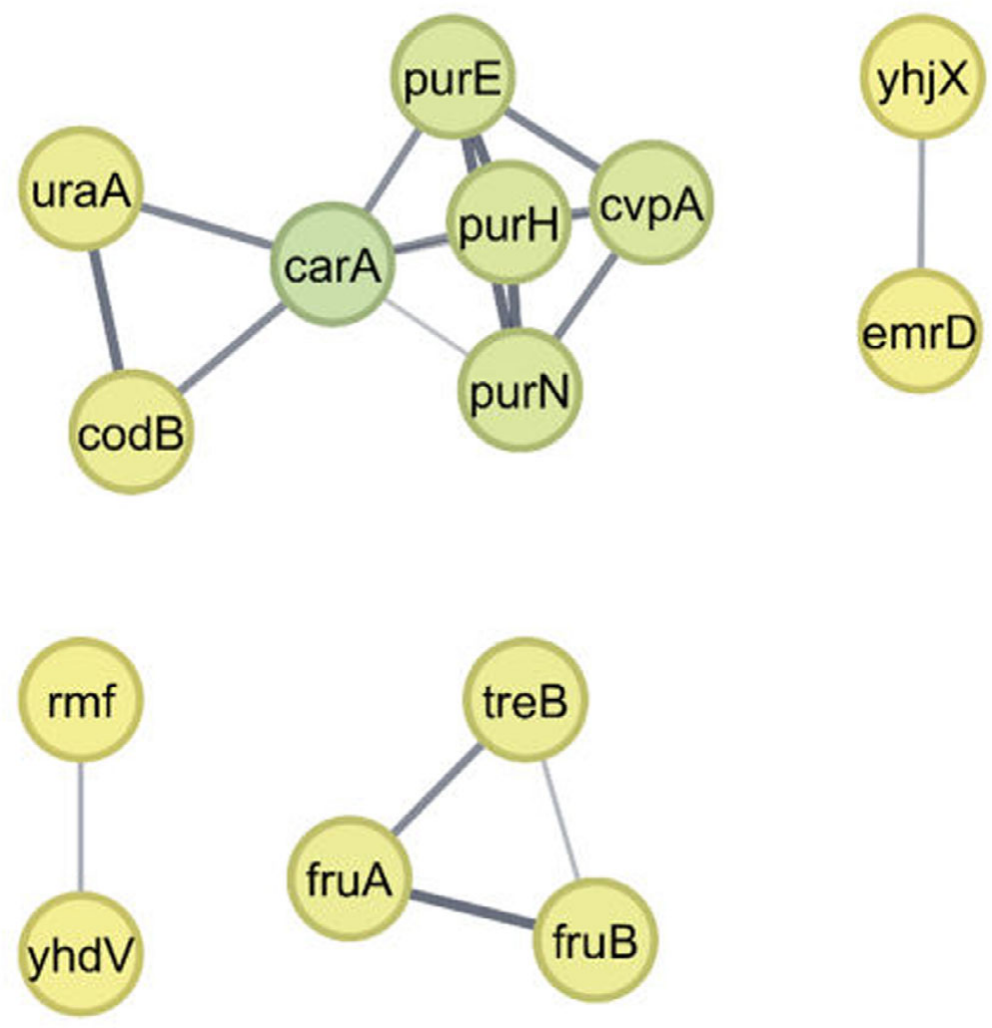
Protein–protein interaction network of downregulated genes under acidic pH (pH 4). The network consists of 27 nodes (proteins) and 18 edges (interactions), with two significant sub‐clusters identified by MCODE analysis (medium confidence; cutoff: 0.4). Node colouring reflects the degree of centrality: dark green represents relatively central nodes (degree 4–6), light green shows moderately connected nodes (degree 2–3), and yellow indicates peripheral nodes (degree < 2).

In contrast, the downregulated protein network, shown in Fig. 5 and detailed in Table S4B, consisted of 27 nodes and 18 edges. According to MCODE analysis, this network was subdivided into two main subclusters. The central subcluster included the proteins CarA, UraA, CodB, PurE, PurH, PurN and CvpA and displayed the highest degree of centrality, ranging from 2 to 6. CarA was the most central (dark green node), while the others were indicated by lighter green nodes. The proteins in this subcluster are involved in purine and pyrimidine biosynthesis. In the purine biosynthesis pathway, purE encodes N^5^‐carboxyaminoimidazole ribonucleotide mutase, which catalyses the conversion of N^5^‐CAIR to 4‐carboxy‐5‐aminoimidazole ribonucleotide [112]; purH encodes a bifunctional AICAR transformylase/IMP cyclohydrolase, responsible for the final two steps of de novo purine biosynthesis [114]; and purN encodes GAR transformylase, which catalyses the third step in the same pathway [115]. For pyrimidine biosynthesis, *carA* encodes the amidotransferase component involved in l‐arginine and pyrimidine biosynthesis [116]; *uraA* encodes a uracil transporter responsible for uracil uptake [117]; and *codB* encodes cytosine permease, which facilitates cytosine import [118]. The downregulation of these genes is consistent with prior findings indicating that purine and pyrimidine metabolism is suppressed under acid stress conditions in *E. coli* [116]. The last protein in this subcluster was the colicin V encoded by *cvpA* [119], which is located upstream of *purF* and is repressed by PurR, the main transcriptional repressor of the purine biosynthetic pathway [120]. The second subcluster, shown as green nodes with a degree of centrality of 2, included FruA, FruB and TreB. These proteins are involved in carbohydrate transport and metabolism, particularly related to fructose and trehalose uptake and processing.

### Reverse transcription‐quantitative polymerase chain reaction (RT‐qPCR) validation

**Fig. 6:**
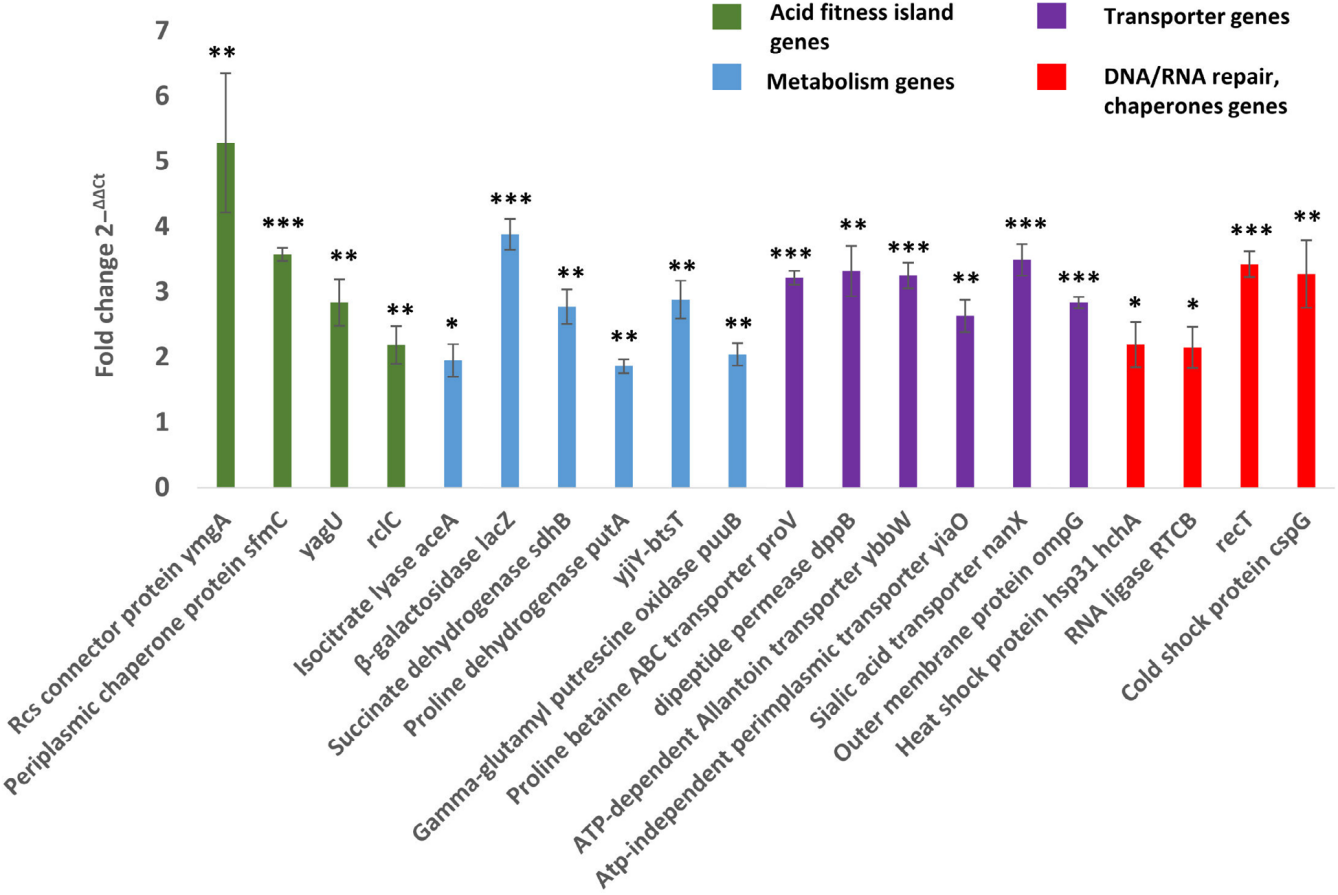
Reverse transcription‐quantitative polymerase chain reaction (RT‐qPCR) validation of 20 selected differentially expressed genes, representing different gene groups. Acid fitness island genes (green), metabolism genes (blue), transporter genes (purple), and DNA/RNA repair and chaperone genes (red). The 16S rRNA gene was used as endogenous control. Data show mean fold change (±SD) in LEA‐K‐expressing *E. coli* BL21 (DE3) relative to the non‐expressing control both grown under acidic conditions pH 4, with three biological replicates (independent cultures) and three technical replicates (repeat assays) per sample. Results were confirmed statistically using *t‐*test, *P < 0.05, **P < 0.01, ***P < 0.001.

To validate the RNA‐Seq results, RT‐qPCR was conducted on a selected 20 differentially expressed genes representing the main functional gene groups including DNA/RNA repair and chaperone, acid responsive genes, metabolism and transporter gene groups. As shown in Fig. 6, the LEA‐K‐expressing *E. coli* BL21 (DE3) showed significant upregulation of the examined genes compared with the control lacking the LEA‐K expression, when both were grown at pH4 in terms of fold change. The RT‐qPCR results were comparable with the RNA‐Seq transcriptomic patterns. However, some genes showed a slightly higher differential expression such as a RcsB C connector gene and this could be due to the difference in sensitivity between the RT‐qpcr and illumina‐sequencing techniques.

### Molecular docking simulation

To elucidate how the LEA‐K peptide confers acid tolerance, we proposed that it might directly engage with bacterial stress response systems, especially TCSs. Molecular docking simulations assessed LEA‐K's binding affinity for acid stress‐related TCS proteins (Rcs, BtsS/BtsR, EnvZ/OmpR). Data illustrated in (Table 2), revealed that LEA‐K exhibited differential binding affinities against the investigated TCS proteins. The docking scores ranged from −114.63 to −158.06, with RcsD exhibiting the most favourable interaction, while the binding free energy (Δ*G*) ranged from −14.6 to −16.5 kcal·mol^−1^. The most favourable energetics was obtained with the BtsR recording Δ*G* of −16.5. Moreover, the ligand efficiency values ranged from −0.143 to −0.159. These results suggest that LEA‐K is capable of forming stable interactions with core components of multiple TCSs.

**Table 2:** Potential interactions of LEA‐K against two‐component systems regulators.

Protein	Docking score	∆*G* (kcal·mol^−1^)	Ligand efficiency
RcsB	−114.63	−14.6	−0.143
RcsD	−158.06	−15.3	−0.147
RcsC	−144.13	−15.8	−0.152
BtsS	−132.96	−15.6	−0.150
BtsR	−137.75	−16.5	−0.159
OmpR	−134.35	−15.1	−0.146
EnvZ	−146.79	−14.9	−0.144

**Fig. 7:**
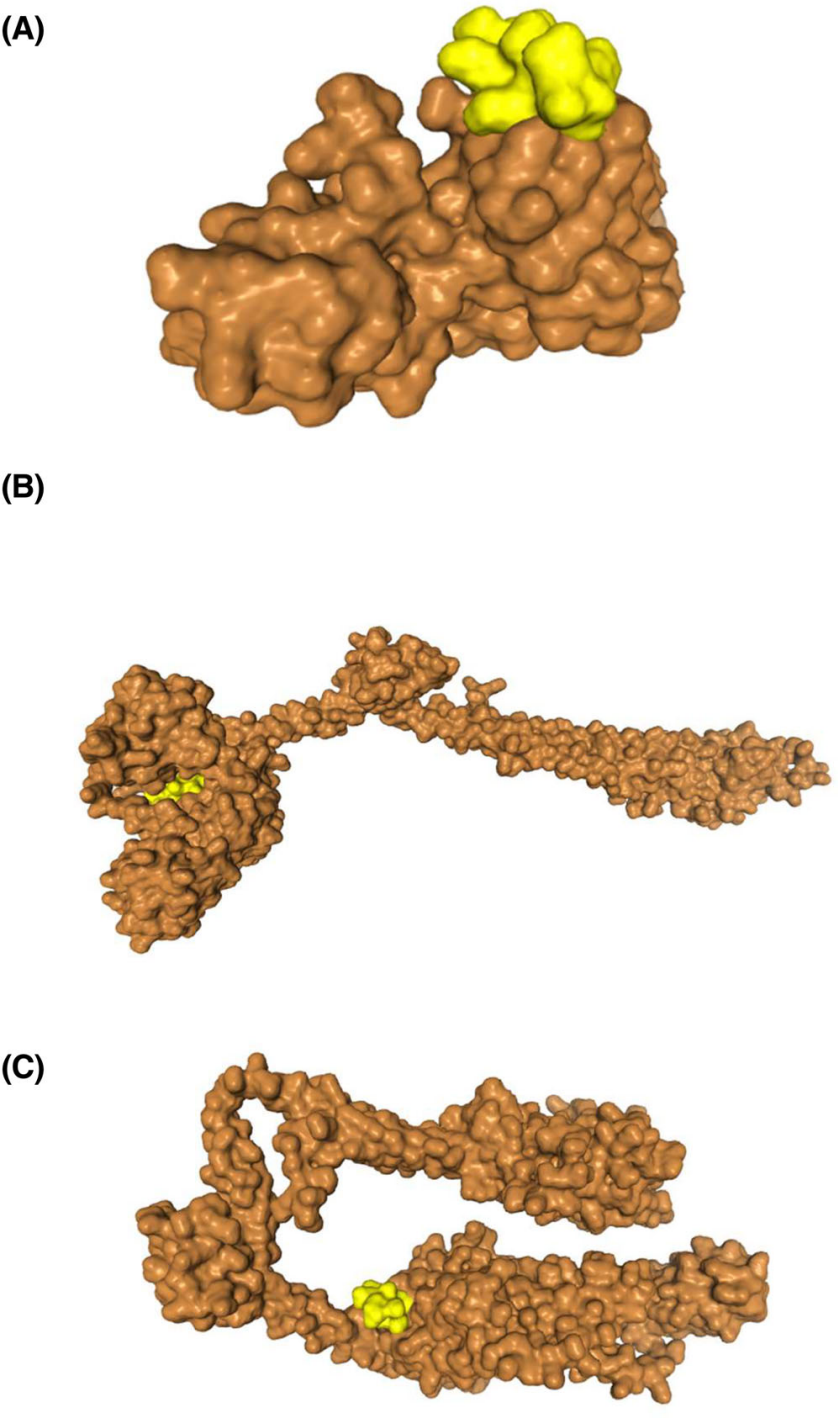
Predicted docking interactions between LEA‐K peptide (ligand) and reactive chlorine species (Rcs) two‐component system regulators (receptors). The LEA‐K peptide structure was predicted using pep‐fold4, followed by molecular docking with Rcs regulators (RcsB/C/D) via HDock with default parameters. (A) LEA‐K (yellow) with RcsB (DNA‐binding response regulator, brown); (B) LEA‐K with RcsC (hybrid sensor kinase, brown); (C) LEA‐K with RcsD (phosphotransfer protein, brown). Top‐scoring poses were selected based on clustering and binding energy scores, with complexes visualised using chimerax.

Prior reports demonstrated the crucial role of the Rcs phosphorelay system in sensing and responding to environmental stresses, including acid and oxidative stress [121]. This pathway involves sequential phosphorylation, beginning with autophosphorylation of RcsC, followed by phosphate transfer to RcsD, and finally to RcsB, the response regulator which modulates gene expression [121, 122]. Based on molecular docking analysis, LEA‐K peptide could interact with the RcsB, RcsC and RcsD (Fig. 7), thereby influencing this signaling cascade. Supporting this hypothesis, transcriptome data also indicated upregulation of the RcsB‐C connector, implying functional activation of the Rcs system in LEA‐K‐expressing *E. coli* under acid stress.

**Fig. 8:**
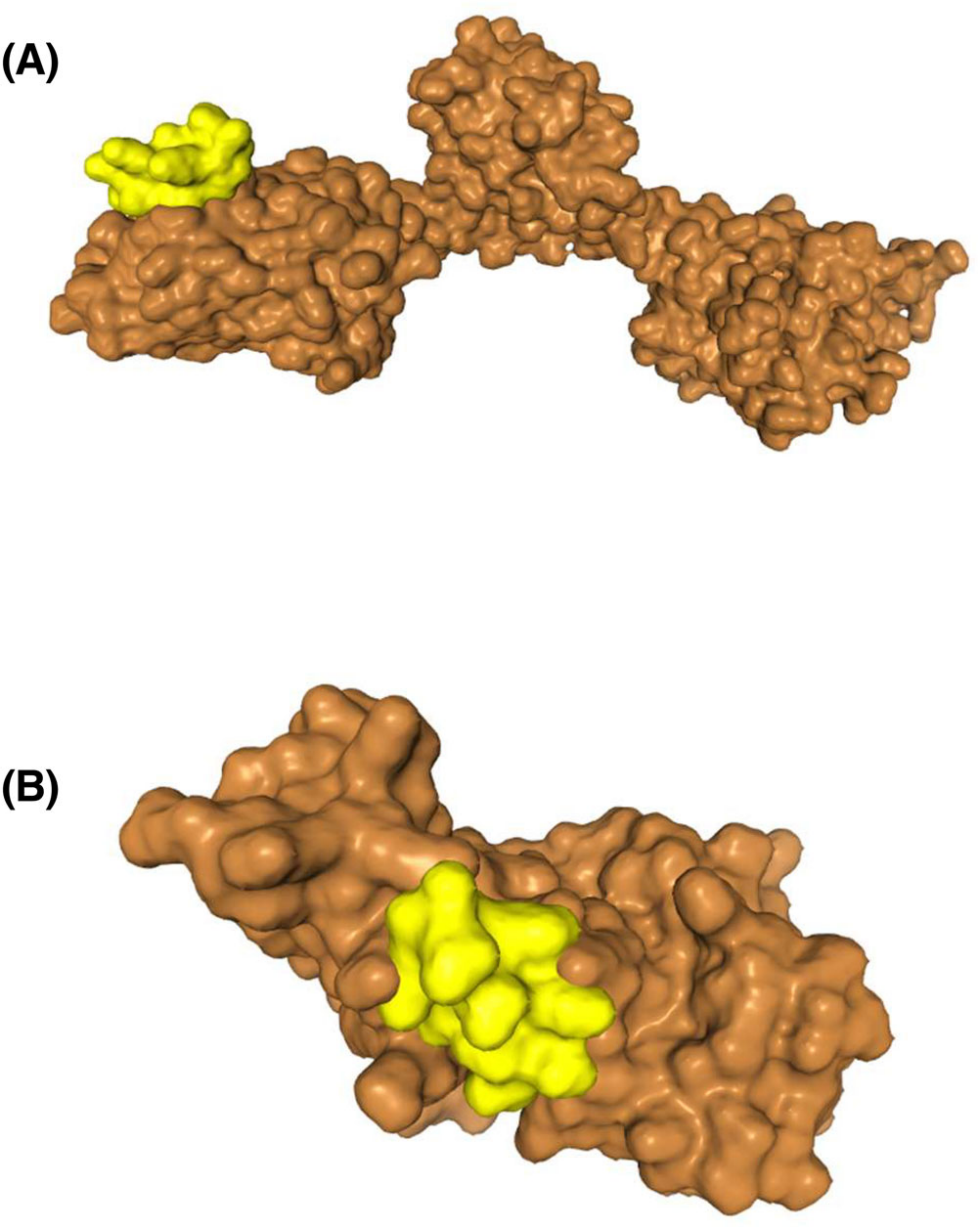
Predicted docking interactions between LEA‐K peptide (ligand) and BtsS/BtsR two‐component system regulators (receptors). The LEA‐K peptide structure was predicted using pep‐fold4, followed by molecular docking with BtsS/BtsR regulators via HDock with default parameters. (A) LEA‐K (yellow) with BtsS (histidine kinase sensor, brown); (B) LEA‐K with BtsR (DNA‐binding response regulator, brown). Top‐scoring presentations were selected based on clustering and binding energy scores, with complexes visualised using chimerax.

**Fig. 9:**
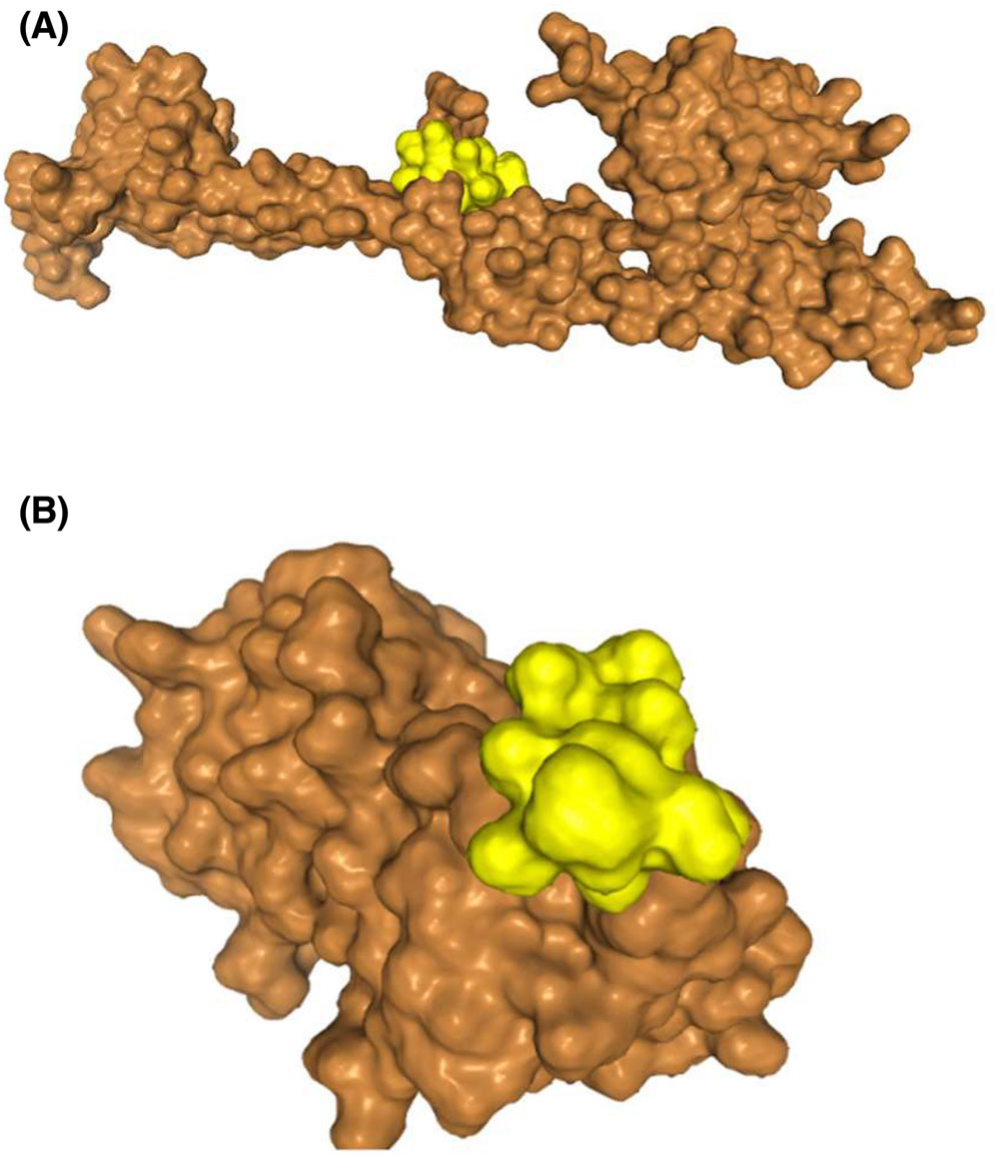
Predicted docking interactions between LEA‐K peptide (ligand) and the EnvZ/OmpR two‐component system regulators (receptors). The LEA‐K peptide structure was predicted using pep‐fold4, followed by molecular docking with EnvZ/OmpR regulators via HDock with default parameters. (A) LEA‐K (yellow) with EnvZ (histidine kinase sensor, brown); (B) LEA‐K with OmpR (DNA‐binding response regulator, brown). Top‐scoring poses were selected based on clustering and binding energy scores, with complexes visualised using chimerax.

The BtsS/R system, well known in gram‐negative bacteria for conferring acid stress tolerance via pyruvate utilisation and proton extrusion [46, 55], was predicted to interact favourably with LEA‐K (Fig. 8). Transcriptomic evidence further supported this interaction, with clear upregulation of *yjiY* and downregulation of *yhjX*, hallmark expression patterns of the newly described Acid Resistance System 6 (AR6) [9]. The EnvZ/OmpR TCS, which is an efficient system in tolerating acid and osmotic stressors via outer membrane porin regulation [123, 124], also exhibited a potential interaction with LEA‐K in docking simulations (Fig. 9). LEA‐K binding to both EnvZ and OmpR may account for transcriptomic shifts observed in porin and transporter gene expression, suggesting an additional pathway through which LEA‐K modulates bacterial physiology under acid stress.

These docking predictions however suggest a potential causal mechanism through direct physical interaction of LEA‐K with investigated TCS components to modulate its activity, potentially explaining the observed gene expression shifts. It is important to emphasise that computational models alone cannot confirm functional interactions. The calculated binding affinities and favourable energetics, though compelling, represent only a predictive framework for LEA‐K's mechanism.

## Materials and methods

### Peptide design

LEA‐K peptide was designed based on the original 11‐mer repetitive sequence found in *Polypedilum vanderplanki* group 3 LEA protein (MDAKDGLKEKAGE), with certain amino acids substitutions generating the following sequence: MDAKDKLKEKAKE [21].

### Cell culture

*E. coli* BL21 (DE3) were transformed with a recombinant pRSF vector encoding the LEA‐K peptide using the heat shock method. Transformants were selected on a Luria‐Bertani (LB) agar plate supplemented with 50 μg·mL^−1^ kanamycin and incubated overnight at 37 °C, and recombinants were subcultured at 37 °C for 14 h in LB broth containing 50 μg·mL^−1^ kanamycin. The cultures were diluted 100‐fold with fresh LB medium and incubated at 37 °C for 2–3 h until the optical density at 600 nm (OD_600_) was 0.5 (mid‐log phase). Peptide expression was induced by adding IPTG to a final concentration of 0 (for control) and 0.1 mm (for treatment), followed by incubation for 4 h at 37 °C with shaking at 120 r.p.m., as previously described [21].

### Acid resistance and growth capacity assay under low pH stress

Cells were harvested by centrifugation at 1500 ***g*** for 10 min following the induction. The pellets were re‐suspended in phosphate‐buffered saline (PBS) at a 1 : 10 dilution. A 2‐mL aliquot of each suspension was inoculated into 50 mL of LB medium, adjusted to pH 4 and incubated at 37 °C and 160 r.p.m. for 2 h. Cultures were serially diluted, and 100 μL of the final two dilutions were plated onto LB agar containing 50 μg·mL^−1^ kanamycin. Plates were incubated at 37 °C for 14 h, and colony‐forming units (CFUs) were recorded, for both LEA‐K‐expressing and non‐expressing strains of *E. coli* BL21 (DE3) [21]. Additionally, bacterial viability was assessed using the Microbial Viability Assay Kit‐WST (Dojindo, Kumamoto, Japan), following the manufacturer's instructions. Briefly, after the acid stress treatment for 2 h and resuspension in PBS, 190 μL of each culture was transferred to a 96‐well clear flat‐bottomed plate (Nunclon, Thermo Fisher Scientific), followed by the addition of 10 μL of WST reagent (prepared as a 9 : 1 mixture with DMSO). Plate was incubated for 1 h, and the viability was quantified colorimetrically using Varioskan LUX Multimode Microplate Reader (Thermo Fisher Scientific, USA) at 450 nm. Results were confirmed statistically using the *t*‐test.

### ROS quantification

Total intracellular reactive oxygen species (ROS) was quantified for the stressed cultures (LEA‐K‐expressing and non‐expressing BL21 (DE3)) using the ROS Assay Kit‐Highly Sensitive DCFH‐DA (Dojindo, Kumamoto, Japan) following the manufacturer's instructions with some modifications. Following the acid stress treatment and re‐suspension in PBS buffer, normalisation was conducted based on the OD_600_ in a volume of 1 mL which was added in clean sterilised 1.5 mL Eppendorf tubes and centrifuged for 5 min at 4000 r.p.m. 100 μL of the ROS assay working solution (1000‐fold dilution of DCFH‐DA dye in loading buffer) was added for re‐suspending the cells and then the total volume was seeded in the 96‐well black‐bottomed plates (Nunclon; Thermo Fisher Scientific, Roskilde, Denmark) and incubated for 30 min at 37 °C in dark conditions. Fluorescence was then measured using the multimode microplate reader (Thermo Fisher Scientific, Varioskan LUX Multimode Microplate Reader, USA) at 490–520 nm excitation and 510–540 nm emission. *t*‐test was employed for statistical analysis.

### RNA extraction and library preparation

Following the 2 h of acid stress treatment, a total of six samples were used for RNA extraction, three biological replicates for each treatment: *E. coli* BL21‐ (DE3) expressing LEA‐K (induced with 0.1 mm IPTG) and non‐expressing controls (no IPTG). RNA stabilisation was achieved using RNAprotect Bacteria Reagent (Qiagen, Hilden, Germany), followed by RNA purification using RNeasy Mini column (Qiagen) following the manufacturer instructions. The purified RNA was quantified spectrophotometrically using Nano‐drop (Thermo Scientific, USA), and analysed on 1% agarose gel. A total of 2 μg RNA from each sample was submitted to the Macrogen Inc. company (Geumcheon‐gu, Seoul, South Korea) for further processing, including removal of rRNA and tRNA and purification of mRNA, and construction of cDNA libraries for Illumina sequencing. Base calling was performed by Illumina's Real‐Time Analysis (rta) software, and sample demultiplexing was carried out using Illumina's bcl2fastq 2.17 software, based on index sequences. Quality scores (Q30) and total read counts were recorded for each library.

### Transcriptomic analysis

The quality of the obtained raw data from illumina sequencing was assessed using fastq quality control [125], and adapter sequences were trimmed using trimmomatic 0.40 [126]. The RNA‐Seq reads were assembled and mapped based on the reference genome of *E. coli* BL21 (DE3) with accession number CP001509 ver. CP001509.3, using bowtie2 software ver. 2.4.1 [127]. The aligned transcripts were assembled using stringtie software ver. 2.1.4 [128].

### Quantification of gene expression levels

The gene expression levels for each biological replicate were estimated using stringtie software [128]. The cleaned reads were mapped to the reference genome CP001509.3, then read counts for each gene were recorded, followed by normalisation of gene count level based on the FPKM (fragments per kilobase of transcript per million fragments). Gene count matrices were created using the PE python script provided by the stringtie manual. Differential gene expression analysis was performed using both edger [129] and deseq2 [130], Bioconductor r based packages. Only genes identified as differentially expressed by both tools were considered for downstream analysis to reduce false positives and increase confidence [131]. Thresholds were set at a *P*‐value < 0.05 and a log2 fold change ≥ 2. The Benjamini–Hochberg method was used to correct for multiple hypothesis testing.

### Functional annotation of differentially expressed genes

Functional annotation was performed for the differentially expressed genes (DEGs), up‐ and downregulated overlapping gene lists identified by both edger and deseq using eggNOG database ver. 5.0 [132], which utilises blast searches against comprehensive protein databases including the NCBI non‐redundant (Nr) protein, Swiss‐Prot, Protein family (Pfam) Protein sequence analysis and classification (InterPro), Kyoto Encyclopedia of Genes and Genomes (KEGG) and Gene Ontology (GO) with an E‐value cutoff of 1e‐5. The unigenes obtained from the analysis were checked to predict the transcription factors using the deeptfactor [109].

### Gene ontology (GO) classification and enrichment analysis

Gene Ontology (GO) classification of DEGs was conducted using the clusterprofiler package in R Bioconductor. DEGs were categorised into three main GO domains: biological process (BP), cellular component (CC) and molecular function (MF). Gene enrichment analysis was performed to identify significantly enriched GO terms associated with DEGs, providing insights into their biological roles.

### Protein–protein interaction (PPI) network analysis

The protein–protein interaction (PPI) network of the DEGs was constructed using string [113], applying a medium confidence score threshold of 0.4. Visualisation and analysis of the PPI network were conducted using cytoscape [133], allowing for detailed exploration of the relationships and functional associations between the identified proteins.

### RT‐qPCR validation

Quantitative reverse transcription PCR (RT‐qPCR) was conducted to validate the RNA‐Seq data. Three biological replicates and three technical replicates were included for both the control group (LEA‐K, 0 mm IPTG) and the treatment group (LEA‐K, 0.1 mm IPTG). RT‐qPCR was performed using the One Step TB Green PrimeScript PLUS RT‐PCR Kit (Takara, Shiga, Japan) in a total reaction volume of 20 μL, which contained 10 μL of 2× One Step TB Green RT‐PCR Buffer 4, 1.2 μL of Takara Ex Taq HS Mix, 0.4 μL of PrimeScript PLUS RTase Mix, 0.8 μL of 10 μm forward and reverse primers, 0.4 μL of ROX Reference Dye (50×), 2 μL of template RNA and 4.4 μL of RNase‐free water. Amplification was carried out using the StepOne Real‐Time PCR System (Applied Biosystems, Waltham, MA, USA). Primer sequences are listed in Table S5. Expression fold changes were calculated using the $2^{-\Delta \Delta C_{t}}$ method, with 16S rRNA serving as the internal reference gene [134]. Results were confirmed statistically using *t‐*test.

### Molecular docking simulations

The potential LEA‐K peptide interaction with TCSs involved in bacterial acid resistance was investigated using molecular docking simulations. Firstly, the LEA‐K peptide was folded up using pep‐fold4 webserver [135], and the folded‐up shape was used for performing the docking against the investigated two‐component systems regulatory proteins using hdock server [136]. ∆*G* (kcal·mol^−1^) and other related calculations were performed using the kdeep server [137]. The generated complexes were visualised using chimerax.

### Data deposition

The six library RNA‐Seq datasets were available on NCBI Sequence Read Archive (SRA) under the BioProject accession number PRJNA1190213.

## Conclusion

This is the first study to explore the fine molecular mechanism mediated by LEA peptide in tolerating acidic pH. Our findings demonstrate that *E. coli* BL21‐expressing LEA‐K showed enhanced growth capacity under acidic conditions compared with the control not expressing. This observation was supported by the transcriptomic analysis, which showed a substantial differential gene expression in LEA‐K‐expressing bacteria. Overall, the LEA‐K expression was strongly associated with reprogrammed cellular functions at multiple levels, including the metabolic and physiological pathways. In terms of metabolic adaptation, the bacteria showed enhanced capacity in utilising alternative carbon and nitrogen sources and preferential activation of adaptive metabolic routes, for example, the glyoxylate shunt and fatty acid degradation were activated to conserve carbon and minimise acidification. Moreover, other prioritised pathways involved in proton neutralisation and acid reduction, such as γ‐glutamyl cycle which converts the putrescine into GABA were activated along with the pyruvate utilisation, allantoin degradation, and ornithine catabolism.

Beyond metabolic adaptations, the transcriptomic analysis revealed the upregulation of various transporters and porins, indicating enhanced cellular capacity to maintain ionic and pH homeostasis, as well as the genes involved in biofilm formation which may contribute to physical protection against acid stress. To investigate potential regulators controlling the observed differential gene expression pattern, we hypothesised that LEA‐K may interact directly with multiple two‐component regulatory systems, including Rcs (RcsB, RcsC and RcsD), BtsS/BtsR and EnvZ/OmpR using in silico docking simulations. For all investigated TCS the computational analysis predicted favourable binding energetics and interaction modes with LEA‐K, though the binding scores varied. These results suggest—but not provide—potential functional modulation of these signaling pathways via LEA‐K, which may account for the observed transcriptional reprogramming. Future studies should bridge the gap between the transcriptomic patterns and docking predictions by (a) expanding computational modeling to other TCS in *E. coli*, (b) validating predicted interactions through proteomic and phosphoproteomic analysis to identify physical binding partners and phosphorylation changes, and (c) employing direct interaction assays such as bacterial two‐hybrid systems to confirm LEA‐K's binding specificity to target TCS components.

## Conflict of interest

The authors declare no conflict of interest.

## Author contributions

SI acquired funding, KM and SI conceived and designed the project, KM performed experiments, KM performed the bioinformatics analysis, KM and SI analysed and interpreted the data, KM and SI drafted the manuscript, KM and SI revised and read the first draft and approved the final version of the manuscript.

## Supporting information


**Table S1.** Summary of differentially expressed genes (DEGs), categorised by functional groups.

**Table S2.** Summary of differentially expressed genes (DEGs) showing fold changes.

**Table S3.** Summary of gene ontology clustering for the differentially expressed gene (DEG) sets.

**Table S4.** Network cluster analysis and centrality metrics of differentially expressed genes (DEGs).

**Table S5.** List of RT‐qPCR primers used.

## Data Availability

The RNA‐seq data generated in this study are openly available in the NCBI Sequence Read Archive (SRA) under BioProject accession number PRJNA1190213. All other supporting data are included in the Supporting Information of this article.

## References

[1] Leung JYS & McAfee D (2020) Stress across life stages: impacts, responses and consequences for marine organisms. Sci Total Environ 700, 134491. 31629264 10.1016/j.scitotenv.2019.134491

[2] Wang Y & Frei M (2011) Stressed food – the impact of abiotic environmental stresses on crop quality. Agric Ecosyst Environ 141, 271–286.

[3] Msimbira LA & Smith DL (2020) The roles of plant growth promoting microbes in enhancing plant tolerance to acidity and alkalinity stresses. Front Sustain Food Syst 4, 564220.

[4] Qiao M , Hong C , Jiao Y , Hou S & Gao H (2024) Impacts of drought on photosynthesis in major food crops and the related mechanisms of plant responses to drought. Plants 13, 1808. 38999648 10.3390/plants13131808 PMC11243883

[5] Nguyen PT , Nguyen TT , Bui DC , Hong PT , Hoang QK & Nguyen HT (2020) Exopolysaccharide production by lactic acid bacteria: the manipulation of environmental stresses for industrial applications. AIMS Microbiol 6, 451–469. 33364538 10.3934/microbiol.2020027 PMC7755584

[6] Lücke FK & Adams MR (2023) Acids and fermentation. In Food Safety Management: A Practical Guide for the Food Industry, 2nd edn, pp. 439–452. Aspen Publishers, Gaithersburg, MD.

[7] Rossi E , Cimdins A , Lüthje P , Brauner A , Sjöling Å , Landini P & Römling U (2018) “It's a gut feeling” – *Escherichia coli* biofilm formation in the gastrointestinal tract environment. Crit Rev Microbiol 44, 1–30. 28485690 10.1080/1040841X.2017.1303660

[8] Kanjee U & Houry WA (2013) Mechanisms of acid resistance in *Escherichia coli* . Annu Rev Microbiol 67, 65–81. 23701194 10.1146/annurev-micro-092412-155708

[9] Li Z , Huang Z & Gu P (2024) Response of *Escherichia coli* to acid stress: mechanisms and applications—A narrative review. Microorganisms 12, 1774. 39338449 10.3390/microorganisms12091774 PMC11434309

[10] Zhu Z , Ji X , Wu Z , Zhang J & Du G (2018) Improved acid‐stress tolerance of *Lactococcus lactis* NZ9000 and *Escherichia coli* BL21 by overexpression of the anti‐acid component recT. J Ind Microbiol Biotechnol 45, 1091–1101. 30232653 10.1007/s10295-018-2075-8

[11] Dure L & Galau GA (1981) Developmental biochemistry of cottonseed embryogenesis and germination: XIII. Regulation of biosynthesis of principal storage proteins. Plant Physiol 68, 187–194. 16661868 10.1104/pp.68.1.187 PMC425913

[12] Cuming AC (1999) LEA proteins. In Seed Proteins ( Casey R & Shewry PR , eds), pp. 753–780. Kluwer Academic Publishers, Dordrecht.

[13] Hincha DK & Thalhammer A (2012) LEA proteins: IDPs with versatile functions in cellular dehydration tolerance. Biochem Soc Trans 40, 1000–1003. 22988854 10.1042/BST20120109

[14] Furuki T & Sakurai M (2016) Group 3 LEA protein model peptides protect enzymes against desiccation stress. Biochim Biophys Acta 1864, 1237–1243. 27131872 10.1016/j.bbapap.2016.04.012

[15] Hand SC , Menze MA , Toner M , Boswell L & Moore D (2011) LEA proteins during water stress: not just for plants anymore. Annu Rev Physiol 73, 115–134. 21034219 10.1146/annurev-physiol-012110-142203

[16] Liu Y , Chakrabortee S , Li R , Zheng Y & Tunnacliffe A (2011) Both plant and animal LEA proteins act as kinetic stabilisers of polyglutamine‐dependent protein aggregation. FEBS Lett 585, 630–634. 21251910 10.1016/j.febslet.2011.01.020

[17] Chakrabortee S , Meersman F , Schierle GSK , Bertoncini CW , McGee B , Kaminski CF & Tunnacliffe A (2010) Catalytic and chaperone‐like functions in an intrinsically disordered protein associated with desiccation tolerance. Proc Natl Acad Sci U S A 107, 16084–16089. 20805515 10.1073/pnas.1006276107 PMC2941266

[18] Battaglia M & Covarrubias AA (2013) Late Embryogenesis Abundant (LEA) proteins in legumes. Front Plant Sci 4, 45960. 10.3389/fpls.2013.00190 PMC3691520 23805145

[19] Pathak N & Ikeno S (2017) In vivo expression of a short peptide designed from late embryogenesis abundant protein for enhancing abiotic stress tolerance in *Escherichia coli* . Biochem Biophys Res Commun 492, 386–390. 28844676 10.1016/j.bbrc.2017.08.091

[20] Huwaidi A , Pathak N , Syahir A & Ikeno S (2018) *Escherichia coli* tolerance of ultraviolet radiation by in vivo expression of a short peptide designed from late embryogenesis abundant protein. Biochem Biophys Res Commun 503, 910–914. 29928878 10.1016/j.bbrc.2018.06.095

[21] Metwally K & Ikeno S (2020) A short peptide designed from late embryogenesis abundant protein enhances acid tolerance in *Escherichia coli* . Appl Biochem Biotechnol 191, 164–176. 32096062 10.1007/s12010-020-03262-5

[22] Gu S , Wu T , Zhao J , Sun T , Zhao Z , Zhang L , Li J & Tian C (2024) Rewiring metabolic flux to simultaneously improve malate production and eliminate by‐product succinate accumulation by *Myceliophthora thermophila* . J Microbial Biotechnol 17, 1–16. 10.1111/1751-7915.14410 PMC10884987 38298109

[23] Yu P , Chen X & Li P (2017) Enhancing microbial production of biofuels by expanding microbial metabolic pathways. Biotechnol Appl Biochem 64, 606–619. 27507087 10.1002/bab.1529

[24] Ikeno S & Haruyama T (2013) Boost protein expression through co‐expression of LEA‐like peptide in *Escherichia coli* . PLoS One 8, e82824. 24349373 10.1371/journal.pone.0082824 PMC3861450

[25] B‐Bj M , A‐Pm A & Hh M (2010) Role of antioxidant enzymes in bacterial resistance to organic acids. Appl Environ Microbiol 76, 2747–2753. 20305033 10.1128/AEM.02718-09 PMC2863438

[26] Bateman A , Martin MJ , Orchard S , Magrane M , Agivetova R , Ahmad S , Alpi E , Bowler‐Barnett EH , Britto R , Bursteinas B *et al*. (2021) UniProt: the universal protein knowledgebase in 2021. Nucleic Acids Res 49, D480–D489. 33237286 10.1093/nar/gkaa1100 PMC7778908

[27] Kobe B & Kajava AV (2001) The leucine‐rich repeat as a protein recognition motif. Curr Opin Struct Biol 11, 725–732. 11751054 10.1016/s0959-440x(01)00266-4

[28] Petersen L , Bollback JP , Dimmic M , Hubisz M & Nielsen R (2007) Genes under positive selection in *Escherichia coli* . Genome Res 17, 1336–1343. 17675366 10.1101/gr.6254707 PMC1950902

[29] Dong H , Tao W , Gong F , Li Y & Zhang Y (2014) A functional recT gene for recombineering of clostridium. J Biotechnol 173, 65–67. 24384234 10.1016/j.jbiotec.2013.12.011

[30] Wang LYR , Jokinen CC , Laing CR , Johnson RP , Ziebell K & Gannon VPJ (2020) Assessing the genomic relatedness and evolutionary rates of persistent verotoxigenic *Escherichia coli* serotypes within a closed beef herd in Canada. Microb Genom 6, e000376. 32496181 10.1099/mgen.0.000376 PMC7371104

[31] Tanaka N , Meineke B & Shuman S (2011) RtcB, a novel RNA ligase, can catalyze tRNA splicing and HAC1 mRNA splicing in vivo. J Biol Chem 286, 30253–30257. 21757685 10.1074/jbc.C111.274597 PMC3162383

[32] Chakravarty AK , Subbotin R , Chait BT & Shuman S (2012) RNA ligase RtcB splices 3′‐phosphate and 5′‐OH ends via covalent RtcB‐(histidinyl)‐GMP and polynucleotide‐(3′)pp(5′)G intermediates. Proc Natl Acad Sci U S A 109, 6072–6077. 22474365 10.1073/pnas.1201207109 PMC3341019

[33] Mujacic M & Baneyx F (2006) Regulation of *Escherichia coli* hchA, a stress‐inducible gene encoding molecular chaperone Hsp31. Mol Microbiol 60, 1576–1589. 16796689 10.1111/j.1365-2958.2006.05207.x

[34] Mujacic M & Baneyx F (2007) Chaperone Hsp31 contributes to acid resistance in stationary‐phase *Escherichia coli* . Appl Environ Microbiol 73, 1014–1018. 17158627 10.1128/AEM.02429-06 PMC1800746

[35] Pan H , Yang D , Wang Y , Rao L & Liao X (2023) Acid shock protein Asr induces protein aggregation to promote *E. coli* O157:H7 entering viable but non‐culturable state under high pressure carbon dioxide stress. Food Microbiol 109, 104136. 36309439 10.1016/j.fm.2022.104136

[36] Lee C & Park C (2017) Bacterial responses to glyoxal and methylglyoxal: reactive electrophilic species. Int J Mol Sci 18, 169. 28106725 10.3390/ijms18010169 PMC5297802

[37] Vergnes A , Henry C , Grassini G , Loiseau L , El Hajj S , Denis Y , Galinier A , Vertommen D , Aussel L & Ezraty B (2022) Periplasmic oxidized‐protein repair during copper stress in *E. coli*: A focus on the metallochaperone CusF. PLoS Genet 18, e1010180. 35816552 10.1371/journal.pgen.1010180 PMC9302797

[38] Loiseau L , Vergnes A & Ezraty B (2022) Methionine oxidation under anaerobic conditions in *Escherichia coli* . Mol Microbiol 118, 387–402. 36271735 10.1111/mmi.14971 PMC9805139

[39] Uppal S & Jawali N (2015) Cyclic AMP receptor protein (CRP) regulates the expression of cspA, cspB, cspG and cspI, members of cspA family, in *Escherichia coli* . Arch Microbiol 197, 497–501. 25637299 10.1007/s00203-015-1085-4

[40] Cardoza E & Singh H (2022) Involvement of CspC in response to diverse environmental stressors in *Escherichia coli* . J Appl Microbiol 132, 785–801. 34260797 10.1111/jam.15219

[41] Flemming HC & Wingender J (2010) The biofilm matrix. Nat Rev Microbiol 89, 623–633. 10.1038/nrmicro2415 20676145

[42] Samoilova Z , Tyulenev A , Muzyka N , Smirnova G & Oktyabrsky O (2019) Tannic and gallic acids alter redox‐parameters of the medium and modulate biofilm formation. AIMS Microbiol 5, 379–392. 31915750 10.3934/microbiol.2019.4.379 PMC6946639

[43] Little DJ , Pfoh R , Le Mauff F , Bamford NC , Notte C , Baker P , Guragain M , Robinson H , Pier GB , Nitz M *et al*. (2018) PgaB orthologues contain a glycoside hydrolase domain that cleaves deacetylated poly‐β(1,6)‐N‐acetylglucosamine and can disrupt bacterial biofilms. PLoS Pathog 14, e1006998. 29684093 10.1371/journal.ppat.1006998 PMC5933820

[44] Kaval KG & Garsin DA (2018) Ethanolamine utilization in bacteria. MBio 9, e00066‐18. 29463652 10.1128/mBio.00066-18 PMC5821096

[45] Tschowri N , Busse S & Hengge R (2009) The BLUF‐EAL protein YcgF acts as a direct anti‐repressor in a blue‐light response of *Escherichia coli* . Genes Dev 23, 522–534. 19240136 10.1101/gad.499409 PMC2648647

[46] Kannan G , Wilks JC , Fitzgerald DM , Jones BD , Bondurant SS & Slonczewski JL (2008) Rapid acid treatment of *Escherichia coli*: transcriptomic response and recovery. BMC Microbiol 8, 37. 18302792 10.1186/1471-2180-8-37 PMC2270276

[47] Korea CG , Badouraly R , Prevost MC , Ghigo JM & Beloin C (2010) *Escherichia coli* K‐12 possesses multiple cryptic but functional chaperone–usher fimbriae with distinct surface specificities. Environ Microbiol 12, 1957–1977. 20345943 10.1111/j.1462-2920.2010.02202.x

[48] Chingcuanco F , Yu Y , Kus JV , Que L , Lackraj T , Lévesque CM & Foster DB (2012) Identification of a novel adhesin involved in acid‐induced adhesion of enterohaemorrhagic *Escherichia coli* O157:H7. Microbiology 158, 2399–2407. 22767547 10.1099/mic.0.056374-0

[49] Crecencio RB , Brisola MC , Bitner D , Frigo A , Rampazzo L , Borges KA , Furian TQ , Salle CTP , Moraes HLS , Faria GA *et al*. (2020) Antimicrobial susceptibility, biofilm formation and genetic profiles of *Escherichia coli* isolated from retail chicken meat. Infect Genet Evol 84, 104355. 32389829 10.1016/j.meegid.2020.104355

[50] He YZ , Xu Y , Sun J , Le Gao B , Li G , Zhou YF , Lian XL , Fang LX , Liao XP , Mediavilla JR *et al*. (2021) Novel plasmid‐borne fimbriae‐associated gene cluster participates in biofilm formation in *Escherichia coli* . Microb Drug Resist 27, 1624–1632. 34077284 10.1089/mdr.2020.0512

[51] Hayes ET , Wilks JC , Sanfilippo P , Yohannes E , Tate DP , Jones BD , Radmacher MD , BonDurant SS & Slonczewski JL (2006) Oxygen limitation modulates pH regulation of catabolism and hydrogenases, multidrug transporters, and envelope composition in *Escherichia coli* K‐12. BMC Microbiol 6, 89. 17026754 10.1186/1471-2180-6-89 PMC1626474

[52] Parker BW , Schwessinger EA , Jakob U & Gray MJ (2013) The RclR protein is a reactive chlorine‐specific transcription factor in *Escherichia coli* . J Biol Chem 288, 32574–32584. 24078635 10.1074/jbc.M113.503516 PMC3820890

[53] Sultana S , Crompton ME , Meurer K , Jankiewicz O , Morales GH , Johnson C , Horbach E , Hoffmann KP , Kr P , Shah R *et al*. (2022) Redox‐mediated inactivation of the transcriptional repressor RcrR is responsible for Uropathogenic *Escherichia coli*'s increased resistance to reactive chlorine species. MBio 13, e0192622. 36073817 10.1128/mbio.01926-22 PMC9600549

[54] Crompton ME , Gaessler LF , Tawiah PO , Polzer L , Camfield SK , Jacobson GD , Naudszus MK , Johnson C , Meurer K , Bennis M *et al*. (2023) Expression of RcrB confers resistance to hypochlorous acid in uropathogenic *Escherichia coli* . J Bacteriol 205, e0006423. 37791752 10.1128/jb.00064-23 PMC10601744

[55] Kristoficova I , Vilhena C , Behr S & Jung K (2018) BtsT, a novel and specific pyruvate/H+ symporter in *Escherichia coli* . J Bacteriol 200, e00599‐17. 29061664 10.1128/JB.00599-17 PMC5738728

[56] Vilhena C , Kaganovitch E , Shin JY , Grünberger A , Behr S , Kristoficova I , Brameyer S , Kohlheyer D & Jung K (2018) A single‐cell view of the BtsSR/YpdAB pyruvate sensing network in *Escherichia coli* and its biological relevance. J Bacteriol 200, e00536‐17. 29038258 10.1128/JB.00536-17 PMC5717152

[57] Zhou S , Zhuang Y , Zhu X , Yao F , Li H , Li H , Zou X , Wu J , Zhou H , Nuer G *et al*. (2019) YhjX regulates the growth of *Escherichia coli* in the presence of a subinhibitory concentration of gentamicin and mediates the adaptive resistance to gentamicin. Front Microbiol 10, 445353. 10.3389/fmicb.2019.01180 PMC6545925 31191496

[58] Gasperotti A , Göing S , Fajardo‐Ruiz E , Forné I & Jung K (2020) Function and regulation of the pyruvate transporter CstA in *Escherichia coli* . Int J Mol Sci 21, 9068. 33260635 10.3390/ijms21239068 PMC7730263

[59] Abdelwahed EK , Hussein NA , Moustafa A , Moneib NA & Aziz RK (2022) Gene networks and pathways involved in *Escherichia coli* response to multiple stressors. Microorganisms 10, 1793. 36144394 10.3390/microorganisms10091793 PMC9501238

[60] Woo JM , Kim JW , Song JW , Blank LM & Park JB (2016) Activation of the glutamic acid‐dependent acid resistance system in *Escherichia coli* BL21(DE3) leads to increase of the fatty acid biotransformation activity. PLoS One 11, e0163265. 27681369 10.1371/journal.pone.0163265 PMC5040553

[61] Gulevich AY , Skorokhodova AY & Debabov VG (2023) The effect of glyoxylate shunt inactivation on biosynthesis of adipic acid through inverted fatty acid β‐oxidation by *Escherichia coli* strains. Appl Biochem Microbiol 59, 267–274.

[62] Hasan CMM & Shimizu K (2008) Effect of temperature up‐shift on fermentation and metabolic characteristics in view of gene expressions in *Escherichia coli* . Microb Cell Fact 7, 35. 19055729 10.1186/1475-2859-7-35 PMC2634768

[63] Zhao H , Zhou F , Xing Q , Cao Z , Liu J & Zhu G (2018) The soluble transhydrogenase UdhA affecting the glutamate‐dependent acid resistance system of *Escherichia coli* under acetate stress. Biol Open 7, bio031856. 30201831 10.1242/bio.031856 PMC6176936

[64] Lazar N , Fay A , Nandakumar M , Boyle KE , Xavier J , Rhee K & Glickman MS (2017) Control of biotin biosynthesis in mycobacteria by a pyruvate carboxylase dependent metabolic signal. Mol Microbiol 106, 1018–1031. 29052269 10.1111/mmi.13865 PMC5916780

[65] Qin J , Guo H , Wu X , Ma S , Zhang X , Yang X , Liu B , Feng L , Liu H & Huang D (2024) Characterization of mild acid stress response in an engineered acid‐tolerant *Escherichia coli* strain. Microorganisms 12, 1565. 39203406 10.3390/microorganisms12081565 PMC11356199

[66] Shimizu K (2013) Metabolic regulation of a bacterial cell system with emphasis on *Escherichia coli* metabolism. ISRN Biochem 2013, 645983. 25937963 10.1155/2013/645983 PMC4393010

[67] Ahn S , Jung J , Jang IA , Madsen EL & Park W (2016) Role of glyoxylate shunt in oxidative stress response. J Biol Chem 291, 11928–11938. 27036942 10.1074/jbc.M115.708149 PMC4882458

[68] Severi E , Müller A , Potts JR , Leech A , Williamson D , Wilson KS & Thomas GH (2008) Sialic acid mutarotation is catalyzed by the *Escherichia coli* β‐propeller protein YjhT. J Biol Chem 283, 4841–4849. 18063573 10.1074/jbc.M707822200

[69] Bell A , Severi E , Owen CD , Latousakis D & Juge N (2023) Biochemical and structural basis of sialic acid utilization by gut microbes. J Biol Chem 299, 102989. 36758803 10.1016/j.jbc.2023.102989 PMC10017367

[70] Horne CR , Venugopal H , Panjikar S , Wood DM , Henrickson A , Brookes E , North RA , Murphy JM , Friemann R , Griffin MDW *et al*. (2021) Mechanism of NanR gene repression and allosteric induction of bacterial sialic acid metabolism. Nat Commun 12, 1988. 33790291 10.1038/s41467-021-22253-6 PMC8012715

[71] Zhang M , White TA , Schuermann JP , Baban BA , Becker DF & Tanner JJ (2004) Structures of the *Escherichia coli* PutA proline dehydrogenase domain in complex with competitive inhibitors. Biochemistry 43, 12539–12548. 15449943 10.1021/bi048737e PMC3727243

[72] Zhang L , Alfano JR & Becker DF (2015) Proline metabolism increases katG expression and oxidative stress resistance in *Escherichia coli* . J Bacteriol 197, 431–440. 25384482 10.1128/JB.02282-14 PMC4285992

[73] Kitko RD , Wilks JC , Garduque GM & Slonczewski JL (2010) Osmolytes contribute to pH homeostasis of *Escherichia coli* . PLoS One 5, e10078. 20386696 10.1371/journal.pone.0010078 PMC2851621

[74] Maurer LM , Yohannes E , Bondurant SS , Radmacher M & Slonczewski JL (2005) pH regulates genes for flagellar motility, catabolism, and oxidative stress in *Escherichia coli* K‐12. J Bacteriol 187, 304–319. 15601715 10.1128/JB.187.1.304-319.2005 PMC538838

[75] Ro C , Cashel M & Fernández‐Coll L (2021) The secondary messenger ppGpp interferes with cAMP‐CRP regulon by promoting CRP acetylation in *Escherichia coli* . PLoS One 16, e0259067. 34705884 10.1371/journal.pone.0259067 PMC8550359

[76] Schneider BL & Reitzer L (2012) Pathway and enzyme redundancy in putrescine catabolism in *Escherichia coli* . J Bacteriol 194, 4080–4088. 22636776 10.1128/JB.05063-11 PMC3416515

[77] Zhao J , Chen K , Palsson BO & Yang L (2024) StressME: unified computing framework of *Escherichia coli* metabolism, gene expression, and stress responses. PLoS Comput Biol 20, e1011865. 38346086 10.1371/journal.pcbi.1011865 PMC10890762

[78] Wang S , Fang Y , Wang Z , Zhang S , Wang L , Guo Y & Wang X (2021) Improving l‐threonine production in *Escherichia coli* by elimination of transporters ProP and ProVWX. Microb Cell Fact 20, 58. 33653345 10.1186/s12934-021-01546-x PMC7927397

[79] Guerra PR , Herrero‐Fresno A , Ladero V , Redruello B , Dos Santos TP , Spiegelhauer MR , Jelsbak L & Olsen JE (2018) Putrescine biosynthesis and export genes are essential for normal growth of avian pathogenic *Escherichia coli* . BMC Microbiol 18, 226. 30587122 10.1186/s12866-018-1355-9 PMC6307189

[80] Schumacher K , Braun D , Kleigrewe K & Jung K (2024) Motility‐activating mutations upstream of flhDC reduce acid shock survival of *Escherichia coli* . Microbiol Spectr 12, e0054424. 38651876 10.1128/spectrum.00544-24 PMC11237407

[81] Baharuddin B (2024) The impact of fructose consumption on human health: effects on obesity, hyperglycemia, diabetes, uric acid, and oxidative stress with a focus on the liver. Cureus 16, e70095. 39355469 10.7759/cureus.70095 PMC11444807

[82] Du B , Yang L , Lloyd CJ , Fang X & Palsson BO (2019) Genome‐scale model of metabolism and gene expression provides a multi‐scale description of acid stress responses in *Escherichia coli* . PLoS Comput Biol 15, e1007525. 31809503 10.1371/journal.pcbi.1007525 PMC6897400

[83] Zhu C , Jiang X , Zhang Y , Lin J , Fu S & Gong H (2015) Improvement of 1,3‐propanediol production in *Klebsiella pneumoniae* by moderate expression of puuC (encoding an aldehyde dehydrogenase). Biotechnol Lett 37, 1783–1790. 25957564 10.1007/s10529-015-1851-z

[84] Critzer FJ , D'Souza DH , Saxton AM & Golden DA (2010) Increased transcription of the phosphate‐specific transport system of *Escherichia coli* O157:H7 after exposure to sodium benzoate. J Food Prot 73, 819–824. 20501031 10.4315/0362-028x-73.5.819

[85] Lamarche MG , Dozois CM , Daigle F , Caza M , Curtiss R , Dubreuil JD & Harel J (2005) Inactivation of the Pst system reduces the virulence of an avian pathogenic *Escherichia coli* O78 strain. Infect Immun 73, 4138–4145. 15972503 10.1128/IAI.73.7.4138-4145.2005 PMC1168596

[86] Spira B , Aguena M , De Castro Oliveira JV & Yagil E (2010) Alternative promoters in the Pst operon of *Escherichia coli* . Mol Genet Genomics 284, 489–498. 20963440 10.1007/s00438-010-0584-x

[87] Freeman SA , Grinstein S & Orlowski J (2023) Determinants, maintenance, and function of organellar pH. Physiol Rev 103, 515–606. 35981302 10.1152/physrev.00009.2022

[88] Fujita D , Tobe R , Tajima H , Anma Y , Nishida R & Mihara H (2021) Genetic analysis of tellurate reduction reveals the selenate/tellurate reductase genes ynfEF and the transcriptional regulation of moeA by NsrR in *Escherichia coli* . J Biochem 169, 477–484. 33136147 10.1093/jb/mvaa120

[89] Sato R , Sawasato K & Nishiyama K i (2019) YnbB is a CdsA paralogue dedicated to biosynthesis of glycolipid MPIase involved in membrane protein integration. Biochem Biophys Res Commun 510, 636–642. 30739787 10.1016/j.bbrc.2019.01.145

[90] Salscheider SL , Jahn A & Schnetz K (2014) Transcriptional regulation by BglJ–RcsB, a pleiotropic heteromeric activator in *Escherichia coli* . Nucleic Acids Res 42, 2999–3008. 24335284 10.1093/nar/gkt1298 PMC3950696

[91] Hirakawa H , Takita A , Sato Y , Hiramoto S , Hashimoto Y , Ohshima N , Minamishima YA , Murakami M & Tomita H (2023) Inactivation of ackA and pta genes reduces GlpT expression and susceptibility to Fosfomycin in *Escherichia coli* . Microbiol Spectr 11, e0506922. 37199605 10.1128/spectrum.05069-22 PMC10269713

[92] Schumacher K , Gelhausen R , Kion‐Crosby W , Barquist L , Backofen R & Jung K (2023) Ribosome profiling reveals the fine‐tuned response of *Escherichia coli* to mild and severe acid stress. mSystems 8, e0103723. 37909716 10.1128/msystems.01037-23 PMC10746267

[93] Rodionova IA , Gao Y , Monk J , Hefner Y , Wong N , Szubin R , Lim HG , Rodionov DA , Zhang Z , Saier MH *et al*. (2022) A systems approach discovers the role and characteristics of seven LysR type transcription factors in *Escherichia coli* . Sci Rep 12, 7274. 35508583 10.1038/s41598-022-11134-7 PMC9068703

[94] Honn M , Lindgren H & Sjöstedt A (2012) The role of MglA for adaptation to oxidative stress of *Francisella tularensis* LVS. BMC Microbiol 12, 14. 22264342 10.1186/1471-2180-12-14 PMC3305382

[95] Du B , Olson CA , Sastry AV , Fang X , Phaneuf PV , Chen K , Wu M , Szubin R , Xu S , Gao Y *et al*. (2020) Adaptive laboratory evolution of *Escherichia coli* under acid stress. Microbiology (Reading) 166, 141–148. 31625833 10.1099/mic.0.000867 PMC7273327

[96] Sampaio MM , Chevance F , Dippel R , Eppler T , Schlegel A , Boos W , Lu YJ & Rock CO (2004) Phosphotransferase‐mediated transport of the osmolyte 2‐O‐α‐Mannosyl‐D‐glycerate in *Escherichia coli* occurs by the product of the mngA (hrsA) gene and is regulated by the mngR (farR) gene product acting as repressor. J Biol Chem 279, 5537–5548. 14645248 10.1074/jbc.M310980200

[97] Utsunomia C , Hori C , Matsumoto K & Taguchi S (2017) Investigation of the *Escherichia coli* membrane transporters involved in the secretion of d‐lactate‐based oligomers by loss‐of‐function screening. J Biosci Bioeng 124, 635–640. 28818426 10.1016/j.jbiosc.2017.06.018

[98] Ishijima S (2021) Magnesium and Mg2+ transport proteins in cells magnesium and Mg2+ transport proteins in cells. J Biol Macromol 21, 55–67.

[99] Dyszel JL , Soares JA , Swearingen MC , Lindsay A , Smith JN & Ahmer BMM (2010) *E. coli* K‐12 and EHEC genes regulated by SdiA. PLoS One 5, e8946. 20126629 10.1371/journal.pone.0008946 PMC2812512

[100] Rodionova IA , Hosseinnia A , Kim S , Goodacre N , Zhang L , Zhang Z , Palsson B , Uetz P , Babu M & Saier MH (2023) *E. coli* allantoinase is activated by the downstream metabolic enzyme, glycerate kinase, and stabilizes the putative allantoin transporter by direct binding. Sci Rep 13, 7345. 37147430 10.1038/s41598-023-31812-4 PMC10163214

[101] de Oliveira MCB & Balan A (2020) The ATP‐binding cassette (ABC) transport Systems in *Mycobacterium tuberculosis*: structure, function, and possible targets for therapeutics. Biology (Basel) 9, 443. 33291531 10.3390/biology9120443 PMC7761784

[102] Delcour AH (2009) Outer membrane permeability and antibiotic resistance. Biochim Biophys Acta 1794, 808–816. 19100346 10.1016/j.bbapap.2008.11.005 PMC2696358

[103] Masi M , Réfregiers M , Pos KM & Pagès JM (2017) Mechanisms of envelope permeability and antibiotic influx and efflux in gram‐negative bacteria. Nat Microbiol 2, 17001. 28224989 10.1038/nmicrobiol.2017.1

[104] Gao Y , Yurkovich JT , Seo SW , Kabimoldayev I , Dräger A , Chen K , Sastry AV , Fang X , Mih N , Yang L *et al*. (2018) Systematic discovery of uncharacterized transcription factors in *Escherichia coli* K‐12 MG1655. Nucleic Acids Res 46, 10682–10696. 30137486 10.1093/nar/gky752 PMC6237786

[105] Akram NA , Shafiq F & Ashraf M (2017) Ascorbic acid‐a potential oxidant scavenger and its role in plant development and abiotic stress tolerance. Front Plant Sci 8, 238088. 10.3389/fpls.2017.00613 PMC5405147 28491070

[106] Wang M , Chan EWC , Wan Y , Wong MH , Wong MH‐Y & Chen S (2021) Active maintenance of proton motive force mediates starvation‐induced bacterial antibiotic tolerance in *Escherichia coli* . Commun Biol 4, 1068. 34521984 10.1038/s42003-021-02612-1 PMC8440630

[107] Auriol C , Bestel‐Corre G , Claude J‐B , Soucaille P & Meynial‐Salles I (2011) Stress‐induced evolution of *Escherichia coli* points to original concepts in respiratory cofactor selectivity. Proc Natl Acad Sci U S A 108, 1278–1283. 21205901 10.1073/pnas.1010431108 PMC3029715

[108] Rensing C & Grass G (2003) *Escherichia coli* mechanisms of copper homeostasis in a changing environment. FEMS Microbiol Rev 27, 197–213. 12829268 10.1016/S0168-6445(03)00049-4

[109] Kim GB , Gao Y , Palsson BO & Lee SY (2021) DeepTFactor: A deep learning‐based tool for the prediction of transcription factors. Proc Natl Acad Sci U S A 118, e2021171118. 33372147 10.1073/pnas.2021171118 PMC7812831

[110] Lehti TA , Bauchart P , Dobrindt U , Korhonen TK & Westerlund‐Wikström B (2012) The fimbriae activator MatA switches off motility in *Escherichia coli* by repression of the flagellar master operon flhDC. Microbiology 158, 1444–1455. 22422754 10.1099/mic.0.056499-0

[111] Duarte‐Velázquez I , de la Mora J , Ramírez‐Prado JH , Aguillón‐Bárcenas A , Tornero‐Gutiérrez F , Cordero‐Loreto E , Anaya‐Velázquez F , Páramo‐Pérez I , Rangel‐Serrano Á , Muñoz‐Carranza SR *et al*. (2022) *Escherichia coli* transcription factors of unknown function: sequence features and possible evolutionary relationships. PeerJ 10, e13772. 35880217 10.7717/peerj.13772 PMC9308461

[112] Finn RD , Coggill P , Eberhardt RY , Eddy SR , Mistry J , Mitchell AL , Potter SC , Punta M , Qureshi M , Sangrador‐Vegas A *et al*. (2016) The Pfam protein families database: towards a more sustainable future. Nucleic Acids Res 44, D279–D285. 26673716 10.1093/nar/gkv1344 PMC4702930

[113] Szklarczyk D , Kirsch R , Koutrouli M , Nastou K , Mehryary F , Hachilif R , Gable AL , Fang T , Doncheva NT , Pyysalo S *et al*. (2023) The STRING database in 2023: protein–protein association networks and functional enrichment analyses for any sequenced genome of interest. Nucleic Acids Res 51, D638–D646. 36370105 10.1093/nar/gkac1000 PMC9825434

[114] Wolan DW , Cheong CG , Greasley SE & Wilson IA (2004) Structural insights into the human and avian IMP Cyclohydrolase mechanism via crystal structures with the bound XMP inhibitor. Biochemistry 43, 1171–1183. 14756553 10.1021/bi030162i

[115] Andersen‐Civil AIS , Ahmed S , Guerra PR , Andersen TE , Hounmanou YMG , Olsen JE & Herrero‐Fresno A (2018) The impact of inactivation of the purine biosynthesis genes, purN and purT, on growth and virulence in uropathogenic *E. coli* . Mol Biol Rep 45, 2707–2716. 30377949 10.1007/s11033-018-4441-z

[116] Shimada T , Hirao K , Kori A , Yamamoto K & Ishihama A (2007) RutR is the uracil/thymine‐sensing master regulator of a set of genes for synthesis and degradation of pyrimidines. Mol Microbiol 66, 744–757. 17919280 10.1111/j.1365-2958.2007.05954.x

[117] Andersen PS , Frees D , Fast R & Mygind B (1995) Uracil uptake in *Escherichia coli* K‐12: isolation of uraA mutants and cloning of the gene. J Bacteriol 177, 2008–2013. 7721693 10.1128/jb.177.8.2008-2013.1995 PMC176843

[118] Tiraby M , Cazaux C , Baron M , Drocourt D , Reynes J‐P & Tiraby G (1998) Concomitant expression of *E. coli* cytosine deaminase and uracil phosphoribosyltransferase improves the cytotoxicity of 5‐fluorocytosine. FEMS Microbiol Lett 167, 41–49. 9785450 10.1111/j.1574-6968.1998.tb13205.x

[119] Warr AR , Giorgio RT & Waldora MK (2020) Genetic analysis of the role of the conserved inner membrane protein CvpA in enterohemorrhagic *Escherichia coli* resistance to deoxycholate. J Bacteriol 203, e00661‐20. 33361192 10.1128/JB.00661-20 PMC8095453

[120] Shaffer CL , Zhang EW , Dudley AG , Dixon BREA , Guckes KR , Breland EJ , Floyd KA , Casella DP , Algood HMS , Clayton DB *et al*. (2017) Purine biosynthesis metabolically constrains intracellular survival of uropathogenic *Escherichia coli* . Infect Immun 85, e00471‐16. 10.1128/IAI.00471-16 PMC5203662 27795353

[121] Meng J , Young G & Chen J (2021) The Rcs system in Enterobacteriaceae: envelope stress responses and virulence regulation. Front Microbiol 12, 627104. 33658986 10.3389/fmicb.2021.627104 PMC7917084

[122] Wall EA , Majdalani N & Gottesman S (2020) IgaA negatively regulates the Rcs Phosphorelay via contact with the RcsD Phosphotransfer protein. PLoS Genet 16, e1008610. 32716926 10.1371/journal.pgen.1008610 PMC7418988

[123] Chakraborty S & Kenney LJ (2018) A new role of OmpR in acid and osmotic stress in *Salmonella* and *E. coli* . Front Microbiol 9, 2656. 30524381 10.3389/fmicb.2018.02656 PMC6262077

[124] Chakraborty S , Winardhi RS , Morgan LK , Yan J & Kenney LJ (2017) Non‐canonical activation of OmpR drives acid and osmotic stress responses in single bacterial cells. Nat Commun 8, 1587. 29138484 10.1038/s41467-017-02030-0 PMC5686162

[125] Andrews S (2010) FastQC: a quality control tool for high throughput sequence data.

[126] Bolger AM , Lohse M & Usadel B (2014) Trimmomatic: a flexible trimmer for Illumina sequence data. Bioinformatics 30, 2114–2120. 24695404 10.1093/bioinformatics/btu170 PMC4103590

[127] Langmead B & Salzberg SL (2012) Fast gapped‐read alignment with bowtie 2. Nat Methods 94, 357–359. 10.1038/nmeth.1923 PMC3322381 22388286

[128] Pertea M , Kim D , Pertea GM , Leek JT & Salzberg SL (2016) Transcript‐level expression analysis of RNA‐seq experiments with HISAT, StringTie and ballgown. Nat Protoc 119, 1650–1667. 10.1038/nprot.2016.095 PMC5032908 27560171

[129] Chen Y , Lun ATL & Smyth GK (2016) From reads to genes to pathways: differential expression analysis of RNA‐seq experiments using Rsubread and the edgeR quasi‐likelihood pipeline. F1000Res 5, 1438. 27508061 10.12688/f1000research.8987.1 PMC4934518

[130] Love MI , Huber W & Anders S (2014) Moderated estimation of fold change and dispersion for RNA‐seq data with DESeq2. Genome Biol 15, 550. 25516281 10.1186/s13059-014-0550-8 PMC4302049

[131] Soneson C & Delorenzi M (2013) A comparison of methods for differential expression analysis of RNA‐seq data. BMC Bioinformatics 14, 7. 23497356 10.1186/1471-2105-14-91 PMC3608160

[132] Huerta‐Cepas J , Szklarczyk D , Heller D , Hernández‐Plaza A , Forslund SK , Cook H , Mende DR , Letunic I , Rattei T , Jensen LJ *et al*. (2019) eggNOG 5.0: a hierarchical, functionally and phylogenetically annotated orthology resource based on 5090 organisms and 2502 viruses. Nucleic Acids Res 47, D309–D314. 30418610 10.1093/nar/gky1085 PMC6324079

[133] Doncheva NT , Morris JH , Gorodkin J & Jensen LJ (2019) Cytoscape StringApp: network analysis and visualization of proteomics data. J Proteome Res 18, 623–632. 30450911 10.1021/acs.jproteome.8b00702 PMC6800166

[134] Schmittgen TD & Livak KJ (2008) Analyzing real‐time PCR data by the comparative CT method. Nat Protoc 36, 1101–1108. 10.1038/nprot.2008.73 18546601

[135] Rey J , Murail S , De Vries S , Derreumaux P & Tuffery P (2023) PEP‐FOLD4: a pH‐dependent force field for peptide structure prediction in aqueous solution. Nucleic Acids Res 51, W432–W437. 37166962 10.1093/nar/gkad376 PMC10320157

[136] Yan Y , Tao H , He J & Huang SY (2020) The HDOCK server for integrated protein–protein docking. Nat Protoc 155, 1829–1852. 10.1038/s41596-020-0312-x 32269383

[137] Jiménez J , Škalič M , Martínez‐Rosell G & De Fabritiis G (2018) KDEEP: protein‐ligand absolute binding affinity prediction via 3D‐convolutional neural networks. J Chem Inf Model 58, 287–296. 29309725 10.1021/acs.jcim.7b00650

