# Supplementary material for: A short peptide derived from late embryogenesis abundant proteins enhances acid tolerance in Escherichia coli via modulation of two‐component regulatory systems
Source: FEBS J. 2025 Sep 27;293(3):781–805. doi: 10.1111/febs.70268 (PMC12871917; doi:10.1111/febs.70268)
Supplement: Supplementary file 5 — Table S5. List of RT‐qPCR primers used. [file FEBS-293-781-s002.pdf]

Table S5. List of RT-qPCR primers

Gene	Forward and Reverse primer	GC%	Tm	Amplicon size
**Rcs connector**	5`TAATGAAATCGGCTGAGGAA3`<br>5`CCTCCTGAATCACTATTGCTA3`	40.0%<br>42.9%	54.4ºC<br>54.5ºC	85 bp
**sfmC**	5` TTTATCCCGCTGATGCTAAA3`<br>5`ATCCACGAATTGACCAGAAA3`	40.0%<br>40.0%	54.82 ºC<br>54.96 ºC	82 bp
**yagU**	5` TTCCGTGGTATGAGAATGTC3`<br>5` CTGTTTCGTAAATCTCTGCG3`	45.0%<br>45.0%	54.90 ºC<br>54.90 ºC	88 bp
**rclC**	5`GCTGGTTAGGTTTATTGGGA3`<br>5`CGGGGTGGTGATTAAAAATG3`	45.0%<br>45.0%	55.01 ºC<br>54.92 ºC	77 bp
**aceA**	5`GAAAAACCTCGACGACAAAA3`<br>5` ATGAACTGGAACTTGTAGCC3`	40.0%<br>45.0%	54.80 ºC<br>55.35 ºC	75 bp
**lacZ**	5` GTATGAACGGTCTGGTCTTT3`<br>5` GCTATGACGGAACAGGTATT3`	45.0%<br>45.0%	55.10 ºC<br>54.95 ºC	137 bp
**sdhB**	5` GACGGGCTGTATGAATGTAT3`<br>5` AAACTTATCGGGATTCCACC3`	45.0%<br>45.0%	55.0 ºC<br>55.1 ºC	78 bp
**putA**	5` CCGAAAGATATTGTGGGCTA3`<br>5` CGTTATTAACCGCACTTTCC3`	45.0%<br>45.0%	54.92 ºC<br>55.11 ºC	79 bp
**yjiY-btsT**	5` CTGTTCCTGATTGTGGTGTA3`<br>5` CTTTGTCAGTACGTTTGTCG3`	45.0%<br>45.0%	55.0 ºC<br>55.0 ºC	88 bp
**puuB**	5` GAAAATCGACTATCGCTGGA3`<br>5` ATAGATGTTGGTATCGAGGC3`	45.0%<br>45.0%	55.39 ºC<br>55.43 ºC	85 bp
**proV**	5`ATTCTCAATAATCCGGCGAA3`<br>5` CGCACTGAATACCTGACTAA3`	40.0%<br>45.0%	54.92 ºC<br>54.92 ºC	75 bp

**dppB**	5`TGAACTCGGCTTAGATAAACC3`<br>5`TTTCATTGAAATGCCTAGATCG3`	42.9%<br>36.4%	55.4 ºC<br>55.0 ºC	88 bp
**ybbW**	5` CGGTCCAATCTTTGACTACA3`<br>5`TTAATCACCACCAGGAACAG3`	45.0%<br>45.0%	55.11 ºC<br>55.0 ºC	78 bp
**yiaO**	5` GTGATCAACAAAGCGAAGTT3`<br>5` GATAGTTACCCGCTTCTTGT3`	40%<br>45%	54.84 ºC<br>54.88 ºC	85 bp
**nanX**	5` GGAACACTGACAGGTACAAT3`<br>5` ATTAGACCGACTACAAAGGC3`	45%<br>45%	55.0 ºC<br>54.9 ºC	80 bp
**ompG**	5`ATGTACGAAATAGAAAACGTCG3`<br>5`CGGCATTAAAATAGACTGAAGG3`	36.5%<br>40.9%	55.0 ºC<br>55.3 ºC	79 bp
**hchA**	5`CGAAGAACTGAAGAAAATGGG3`<br>5`AGTTTACGGTCCTTATGTACTC3`	42.9%<br>40.9%	55.2 ºC<br>55.0 ºC	75 bp
**RTCB**	5` CATCTGATGACCTGTGGATC3`<br>5` GCTGCATGTTGGGTAAATTT3`	50.0%<br>40.0%	55.3 ºC<br>55.1 ºC	75 bp
**recT**	5`GTTACGTCATAAAGCCATGATT3`<br>5`GGCTTCATCCTTGTCATAGATA3`	36.4%<br>40.9%	54.9 ºC<br>55.1 ºC	76 bp
**cspG**	5`GGTTTAACGCAGATAAAGGT3`<br>5`GCAGTGAAATGGACGAAAAC3`	40.0%<br>45.0%	53.61ºC<br>55.88ºC	76 bp
**16srDNA**	5` GTTTTCAGAGATGAGAATGTGC3`<br>5` GACTTAACCCAACATTTCACAA3`	40.9%<br>36.4%	55.7 ºC<br>55.1 ºC	91 bp

Supplementary Tables legends

Table S1: Summary of differentially expressed genes (DEGs), categorized by functional groups. (A) DNA/RNA repair and Chaperones genes, (B) Biofilm formation genes, (C) Acid stress-responsive genes, (D) Metabolism genes, (E) Transporters and porins, and (F) Predicted transcription factors.

Table S2: Summary of differentially expressed genes (DEGs) showing fold changes, and (A) their functional annotation.

Table S3: Summary of gene ontology clustering for the differentially expressed gene (DEG) sets. (A) Biological process, (B) Molecular function, and (C) Cellular component.

Table S4: Network cluster analysis and centrality metrics of differentially expressed genes (DEGs). (A) Upregulated DEGs, (B) Downregulated DEGs.

Table S5: List of RT-qPCR primers used.
